# Enhanced influenza A H1N1 T cell epitope recognition and cross-reactivity to protein-O-mannosyltransferase 1 in Pandemrix-associated narcolepsy type 1

**DOI:** 10.1038/s41467-021-22637-8

**Published:** 2021-04-16

**Authors:** A. Vuorela, T. L. Freitag, K. Leskinen, H. Pessa, T. Härkönen, I. Stracenski, T. Kirjavainen, P. Olsen, O. Saarenpää-Heikkilä, J. Ilonen, M. Knip, A. Vaheri, M. Partinen, P. Saavalainen, S. Meri, O. Vaarala

**Affiliations:** 1 Clinicum, University of Helsinki, Helsinki, Finland; 2 Department of Bacteriology and Immunology, University of Helsinki, Helsinki, Finland; 3 Translational Immunology Research Program, University of Helsinki, Helsinki, Finland; 4 Children’s Hospital, University of Helsinki, and Helsinki University Hospital, Helsinki, Finland; 5 Department of Child Neurology, Oulu University Hospital, Oulu, Finland; 6 Department of Pediatrics, Tampere University Hospital, Tampere, Finland; 7 Immunogenetics Laboratory, Institute of Biomedicine, University of Turku, Turku, Finland; 8 Clinical Microbiology, Turku University Hospital, Turku, Finland; 9 Research Program for Clinical and Molecular Metabolism, University of Helsinki, Helsinki, Finland; 10 Department of Virology, University of Helsinki, Helsinki, Finland; 11 Department of Neurosciences, University of Helsinki, Helsinki, Finland; 12 Helsinki Sleep Clinic, Vitalmed Research Center, Helsinki, Finland

**Keywords:** Inactivated vaccines, Paediatric research, Sleep disorders, Adaptive immunity

## Abstract

Narcolepsy type 1 (NT1) is a chronic neurological disorder having a strong association with HLA-DQB1*0602, thereby suggesting an immunological origin. Increased risk of NT1 has been reported among children or adolescents vaccinated with AS03 adjuvant-supplemented pandemic H1N1 influenza A vaccine, Pandemrix. Here we show that pediatric Pandemrix-associated NT1 patients have enhanced T-cell immunity against the viral epitopes, neuraminidase 175–189 (NA_175–189_) and nucleoprotein 214–228 (NP_214–228_), but also respond to a NA_175–189_-mimic, brain self-epitope, protein-O-mannosyltransferase 1 (POMT1_675–689_). A pathogenic role of influenza virus-specific T-cells and T-cell cross-reactivity in NT1 are supported by the up-regulation of IFN-γ, perforin 1 and granzyme B, and by the converging selection of T-cell receptor TRAV10/TRAJ17 and TRAV10/TRAJ24 clonotypes, in response to stimulation either with peptide NA_175–189_ or POMT1_675–689_. Moreover, anti-POMT1 serum autoantibodies are increased in Pandemrix-vaccinated children or adolescents. These results thus identify POMT1 as a potential autoantigen recognized by T- and B-cells in NT1.

## Abstract (web summary)

Narcolepsy type 1 (NT1) is a severe sleep disorder with strong association to the HLA type DQB1*0602 and increased incidence among children vaccinated with the Influenza A vaccine Pandemrix. Here the authors show that these children develop T and B cell autoimmunity against protein-O-mannosyltransferase 1 via cross-reactivity.

## Introduction

Narcolepsy type 1 (NT1) is a rare, chronic brain disorder, characterized by excessive daytime sleepiness, cataplexy and disturbed nocturnal sleep^1–3^. An exceptionally high association of NT1 with the human leukocyte antigen (HLA) class II allele DQB1*0602, and polymorphisms in the genes encoding the T-cell receptor (TCR) α-chain (TRA), tumor necrosis factor-α (TNF-α) promoter, TNF receptor 2 and P2Y11 receptor, strongly suggest an immunological origin of the disease^4–8^. In particular, T-cell mediated mechanisms leading to impaired hypocretin (HCRT) signaling have been implicated in the development of NT1^4,9–11^. Increased risk of NT1 has been reported from several European countries, among children and adolescents vaccinated during 2009/10 with the AS03 adjuvant-supplemented pandemic H1N1 influenza A vaccine (Pandemrix®)^12–15^. We previously reported that Pandemrix-associated NT1 patients showed enhanced antibody responses to influenza A virus proteins present in Pandemrix vaccine^16^. This suggested that vaccinees who later developed NT1 may also have mounted aberrant T-cell responses to Pandemrix. Further, we hypothesized that CD4+ T-cells primed by influenza A (H1N1) virus peptides cross-react with CNS autoantigens, and orchestrate an immune-mediated attack on the hypocretin neuronal network in NT1.

Here we demonstrate enhanced T helper 1 (Th1)-cell/cytotoxic T-cell responses in Pandemrix-associated NT1 patients against distinct viral NA and NP peptide epitopes. Patient-derived T-cells also respond against a self-epitope in brain-expressed protein-O-mannosyltransferase 1 (POMT1), that represents a peptide mimic of a T-cell epitope of NA strongly recognized in patients. Peripheral blood mononuclear cell (PBMC) gene expression profiles are closely matched, and TCR repertoire analyses detect upregulation of identical clonotypes in response to either NA or POMT1 peptide stimulation, thus supporting molecular mimicry and a pathogenic role of virus-directed, autoreactive T-cell clonotypes in NT1. Finally, vaccinees have increased antibody levels against human POMT1, suggesting that Pandemrix vaccination triggers POMT1 autoimmunity. These findings provide a link between influenza A (H1N1) virus-directed T-cell immunity and the development of autoimmunity in NT1.

## Results

### Influenza A H1N1 virus T-cell epitope screen in Pandemrix-vaccinated HLA-DQ6.2 transgenic mice, and HLA-DQB1*0602 positive individuals

**Fig. 1:**
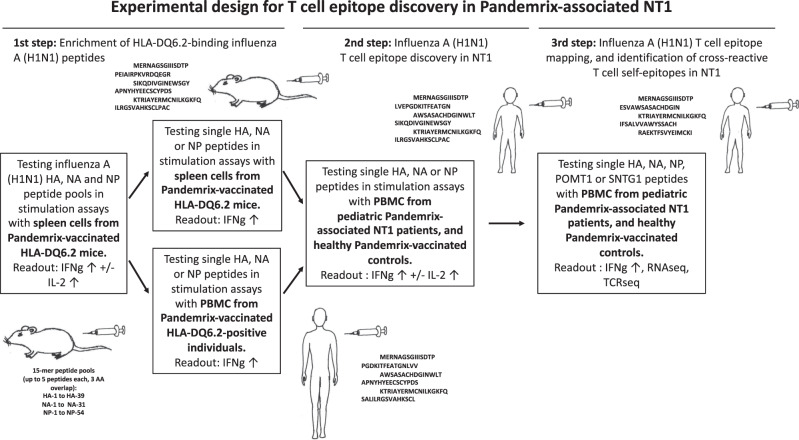
Overall experimental study design for T-cell epitope discovery in Pandemrix-associated NT1. As a first step, influenza A (H1N1) virus HA, NA, and NP peptide T cell recognition was tested with spleen cells from Pandemrix-vaccinated HLA-DQ6.2 mice, restimulated with pools of 5 peptides each (15-mers). Pools that stimulated IFN*-γ* or IL-2 expression were broken up, and single peptides tested either with spleen cells from additional Pandemrix-vaccinated HLA-DQ6.2 mice, or PBMC from Pandemrix-vaccinated HLA-DQB1*0602 positive individuals. As a second step, recognition of single peptides was then tested with PBMC from pediatric Pandemrix-associated NT1 patients, and healthy Pandemrix-vaccinated controls. As a third step, influenza A (H1N1) T cell peptides that showed increased stimulation of IFN*-*γ or IL-2 secretion in patients vs. controls were validated and mapped. Cross-reactive T cell self-epitopes were predicted by BLAST against human proteome, and recognition tested with PBMC from pediatric Pandemrix-associated NT1 patients, and healthy Pandemrix-vaccinated controls.

**Fig. 2:**
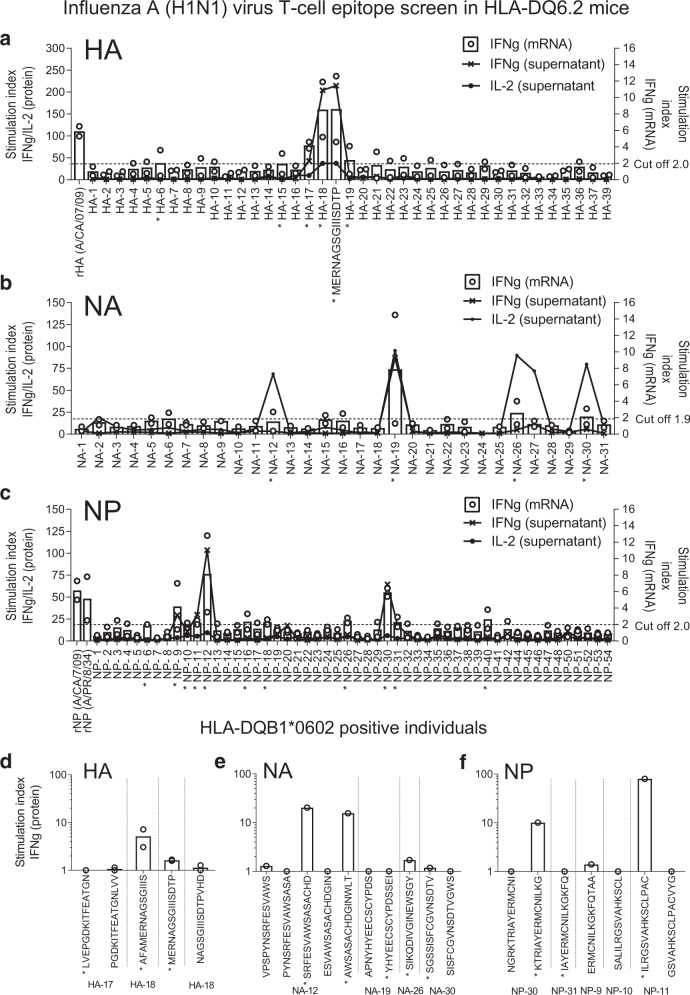
Influenza A H1N1 virus T-cell epitope screen in HLA-DQ6.2 mice, and Pandemrix-vaccinated HLA-DQB1*0602 positive individuals. Spleen cells from Pandemrix-immunized HLA-DQ6.2 mice were stimulated in culture with pools of five overlapping 15-mer peptides each, covering **a** hemagglutinin (HA), **b** neuraminidase (NA) or **c** nucleoprotein (NP) from influenza (A/reassortant/NYMC X-179A (California/07/2009 × NYMC X-157)(H1N1)) vaccine virus used in Pandemrix (pooling cells from 2 × 2 mice, *n* = 2). Recombinant hemagglutinin (rHA) and nucleoprotein (rNP; A/Puerto Rico/8/34 corresponding to the Pandemrix vaccine strain) were used as positive controls. **d**–**f** PBMC from Pandemrix-vaccinated HLA-DQB1*0602 positive individuals (sleep clinic patients without a diagnosis of NT1) were stimulated in culture with single 15-mer peptides, derived from the same vaccine virus strain (*n* = 1–2). The expression of IFN-γ or IL-2 was measured by FMIA (protein) or RT-qPCR (mRNA). Results are expressed as ratios between cytokine concentrations (lines representing means) or relative gene expressions (dots representing single values, bars representing means) measured in peptide-stimulated and negative control samples (stimulation index). An asterisk (*) indicates a pool or single peptide that was selected for further testing.

To map influenza A (H1N1) T-cell epitopes in Pandemrix-associated NT1, 15-mer peptides covering hemagglutinin (HA), NA, and NP of influenza (A/reassortant/NYMC X-179A (California/07/2009 × NYMC X-157)(H1N1)) vaccine virus with 12 amino acid overlap were produced. The experimental design for T-cell epitope discovery is illustrated in Fig. 1. First, we screened T-cell responses to peptide pools (Supplementary Data 1) using spleen cells from Pandemrix-vaccinated HLA-DQ6.2 transgenic mice^17^. Upregulation of interferon-γ (IFN-γ) and/or interleukin-2 (IL-2) mRNA (RT-qPCR) or protein (fluorescent multiplex bead-based immunoassay, FMIA) were used as read-out (Fig. 2a–c). As the second step, T-cell reactivity to single peptides from pools stimulating IFN-γ or IL-2 upregulation was tested in spleen cells from Pandemrix-vaccinated HLA-DQ6.2 mice (Supplementary Fig. 1A–C) and in peripheral blood mononuclear cells (PMBC) from Pandemrix-vaccinated HLA-DQB1*0602 positive individuals (sleep clinic control patients without a diagnosis of NT1; Fig. 2d–f; Supplementary Table 1). Single T-cell peptides from HA, NA or NP that stimulated IFN-γ upregulation either in mice, or in HLA-DQB1*0602 positive individuals, were identified as antigens for subsequent T-cell assays comparing NT1 patients and controls (Supplementary Table 2).

### Identification of influenza A H1N1 virus T-cell epitopes in Pandemrix-associated NT1 patients

**Fig. 3:**
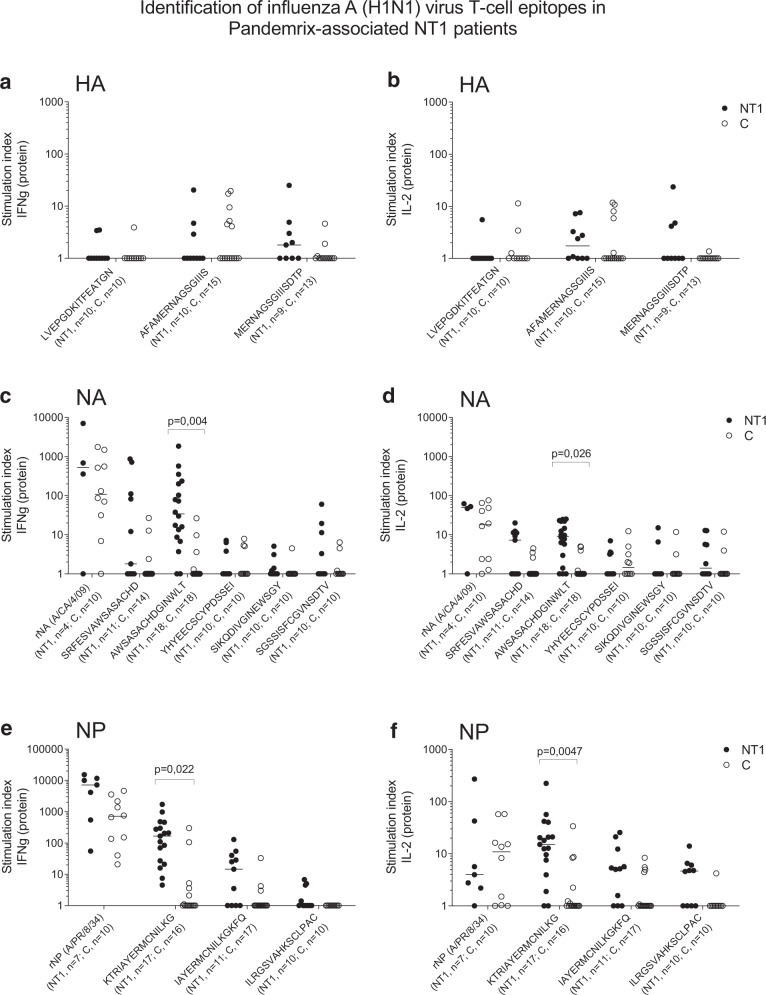
Identification of influenza A H1N1 virus T-cell epitopes in Pandemrix-associated NT1 patients. PBMC from pediatric Pandemrix-associated NT1 patients (NT1) or pediatric Pandemrix-vaccinated healthy controls (C) were stimulated in culture with single 15-mer peptides from influenza (A/reassortant/NYMC X-179A (California/07/2009 × NYMC X-157)(H1N1)) vaccine virus hemagglutinin (HA), neuraminidase (NA) or nucleoprotein (NP) (discovery cohort). Recombinant neuraminidase (rNA) and nucleoprotein (rNP) were used as positive controls. The secretion of IFN-γ (**a**, **c**, **e**) or IL-2 (**b**, **d**, **f**) was measured by FMIA (protein). Results are expressed as the ratio between cytokine concentrations measured in peptide-stimulated and negative control samples (stimulation index). Statistical comparisons between groups were performed, using Kruskal–Wallis and Dunn’s multiple comparisons tests.

To dissect the influenza A (H1N1)-directed T-cell immunity, PBMC from a discovery cohort of well-characterized Finnish pediatric cases of Pandemrix-associated NT1, and from healthy age-matched, Pandemrix-vaccinated control children (Table 1, Supplementary Tables 3A, B), were stimulated with a panel of pre-selected 15-mer peptides from HA, NA or NP (Supplementary Table 2). Interestingly, two viral T-cell epitopes were differentially recognized and induced high IFN-γ and IL-2 responses in Pandemrix-associated NT1 patients in comparison to healthy vaccinees. These epitopes were AWSASACHDGINWLT (NA_178–192_) and KTRIAYERMCNILKGKFQ (NP_214–231_). Epitopes AFAMERNAGSGIIIS (HA_271–285_) and MERNAGSGIIISDTP (HA_274–288_), previously associated with NT1^18^, were also recognized, but the IFN-γ and IL-2 responses were relatively low, and no significant differences between patients and healthy vaccinees were observed (Fig. 3a–f, Supplementary Fig. 2A–F)

**Table 1:** Clinical information on PBMC and plasma sample donors.

	PBMC				Plasma		
	NT1 patients (all)	NT1 patients (Discovery cohort)	NT1 patients (Validation cohort)	Healthy controls	NT1 patients	Healthy vaccinated controls	Healthy unvaccinated controls
Number of subjects	28	20	14	33	47	57	130
Vaccinated with Pandemrix (%)	100	100	100	100	100	100	0
Age at vaccination (years), median (range)	11.5 (5.1–20.9)	11.1 (5.1–20.9)	11.5 (6.9–18.6)	8.5 (4.1–16.4)	11.7 (4.4–16.6)	7.5 (0.9– 14.8)	n/a
Age at vaccination (years), mean	11.8	11.1	12.5	9.0	11.4	8.3	n/a
Age at sampling (years), median (range)	15.1 (7.7–23.1)	13.4 (7.7–23.1)	15.4 (10.9–23.1)	9.8 (5.5–18.7)	13.0 (5.9–17.9)	9.0 (1.4–16.5)	11.0 (4.0–18.0)
Time between vaccination and sampling (days), median	1078	805	1640	527	550	516	n/a
HLA DQB1*06:02 genotype, *N* (%)	28 (100)	21 (100)	14 (100)	14 (42.4)	47 (100)	21 (36.8)	68 (52.3)
Gender, female, *N* (%)	18 (64.3)	11 (52.4)	9 (64.2)	19 (57.6)	28 (59.6)	30 (52.6)	61 (46.9)

**Fig. 4:**
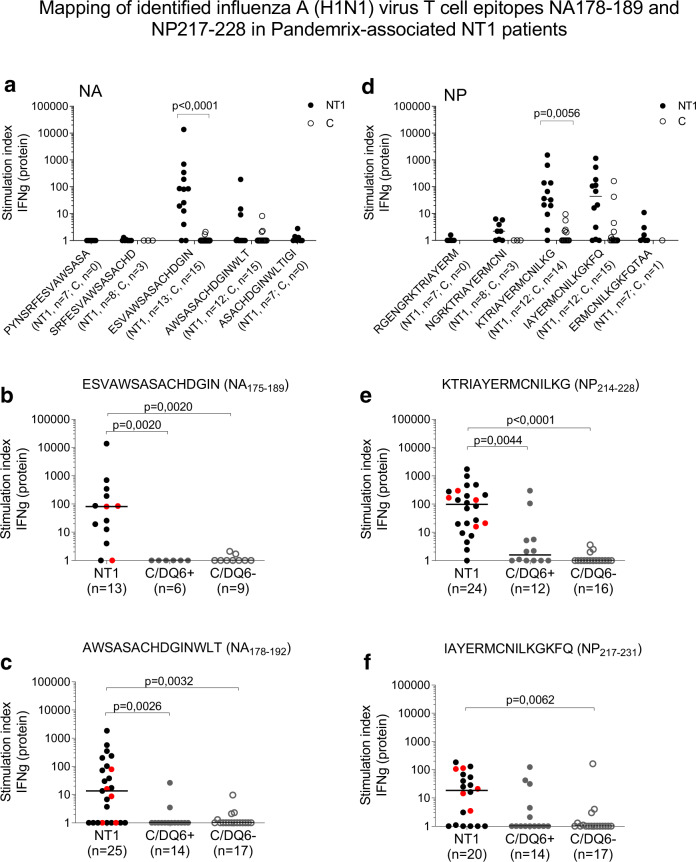
Mapping of identified influenza A H1N1 virus T-cell epitopes in Pandemrix-associated NT1 patients. **a**, **d** PBMC from pediatric Pandemrix-associated NT1 patients (NT1; validation cohort) or pediatric Pandemrix-vaccinated healthy controls (C) were stimulated in culture with overlapping 15-mer peptides from influenza (A/reassortant/NYMC X-179A (California/07/2009 × NYMC X-157)(H1N1)) vaccine virus neuraminidase (NA) or nucleoprotein (NP), as indicated. **b**, **c**, **e**, **f** PBMC from NT1 patients (invariably HLA-DQB1*0602 positive; discovery and validation cohorts combined; HLA-DQB1*0602 homozygous NT1 patients marked with red dots) or HLA-DQB1*0602 positive (C/DQ6+) or negative (C/DQ6−) healthy controls were stimulated with single NA- or NP-derived peptides. The secretion of IFN-γ was measured by FMIA (protein). Results are expressed as the ratio between cytokine concentrations measured in peptide-stimulated and negative control samples (stimulation index). Statistical comparisons between groups were performed, using Kruskal–Wallis and Dunn’s multiple comparisons tests.

Next, the AWSASACHDGINWLT (NA_178–192_) and KTRIAYERMCNILKG (NP_214–228_) epitopes were mapped in a validation cohort of Finnish pediatric Pandemrix-associated NT1 patients, and from healthy age-matched, Pandemrix-vaccinated controls (Table 1, Supplementary Tables 3A, B). Stimulations with overlapping 15-mer peptides in T-cell assays showed that AWSASACHDGIN (NA_178–189_) and IAYERMCNILKG (NP_217–228_) are T-cell core epitopes (Fig. 4a, d, Supplementary Fig. 3A, D). Interestingly, the AWSASACHDGIN (NA_178–189_) epitope includes WSASACHD (NA_179–186_), an influenza A (H1N1) B-cell epitope, predicted by phage display-mimotope variation analysis to react with sera from Pandemrix-associated NT1 patients^19^.

### Differential T-cell responses to influenza A H1N1 epitopes in NT1 patients and HLA-DQB1*0602 positive or negative healthy vaccinees

T cell responses to the epitopes NA_175–189_, NA_178–192_, and NP_214–228_ were stronger in NT1 patients than in either HLA-DQB1*0602 positive or negative controls (combination of NT1 patient discovery and validation cohorts; Fig. 4b, c, e, Supplementary Fig. 3B, C, E). Homozygosity for HLA-DQB1*0602 did not appear to augment responses to these T-cell peptides beyond the levels reached in HLA-DQB1*0602 heterozygous NT1 patients. In contrast, T-cell responses against HA_271–285_ and HA_274–288_ were not increased in NT1 patients in comparison to controls, while responses against NP_217–231_ were significantly enhanced in NT1 patients only when compared to HLA-DQB1*0602 negative, but not positive controls (Supplementary Fig. 4A–D, Fig. 4f, Supplementary Fig. 3F). The results indicated that NT1 patients mounted unusually strong T-cell responses against NA_175–192_ and NP_214–228_ viral epitopes in response to Pandemrix vaccination, and that this was not simply a result of restriction by HLA molecules present on the DRB1*1501-DRB5*0101-DQA1*0102-DQB1*0602 haplotype. These findings were consistent with our previous report of enhanced antibody responses in NT1 patients against viral proteins from Pandemrix, also in comparison to healthy vaccinees carrying the HLA-DQB1*0602 allele^16^.

### Search for human protein sequences with homology to identified influenza A H1N1 T-cell epitopes from NA and NP

To identify possible cross-reactive self-epitopes, we performed basic local alignment of 12-mer peptides against human proteome (blastp). Sequence similarity searches for IAYERMCNILKG (NP_217–228_) and ESVAWSASACHD (NA_175–186_) produced top hits for syntrophin gamma-1 (YEIMCKILK, SNTG1_338–346_; E-value 2.0, 75% query cover, 78% identity) and protein-O-mannosyltransferase 1 (VAWYSSACH, POMT1_681-689_; E-value 0.083, 75% query cover, 78% identity). Both proteins are known to be expressed in the human brain.

### Discovery of a putative influenza A H1N1 virus cross-reactive T-cell self-epitope in Pandemrix-associated NT1 patients

**Fig. 5:**
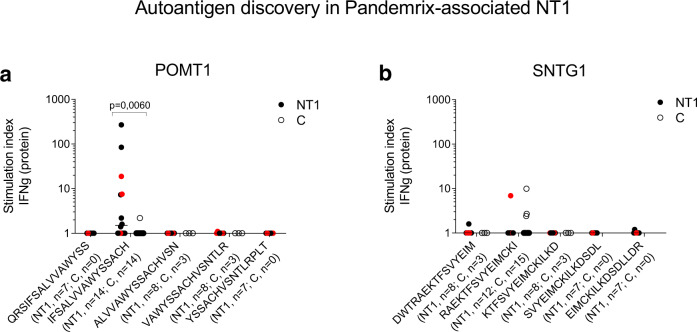
Autoreactive T-cells in Pandemrix-associated NT1. **a**, **b** PBMC from pediatric Pandemrix-associated NT1 patients (NT1) or pediatric Pandemrix-vaccinated healthy controls (C) were stimulated in culture with overlapping 15-mer peptides derived from human protein-O-mannosyltransferase 1 (POMT1) or syntrophin gamma-1 (SNTG1), as indicated. The secretion of IFN-γ was measured by FMIA (protein). HLA-DQB1*0602 homozygous NT1 patients are marked with red dots. Results are expressed as the ratio between cytokine concentrations measured in peptide-stimulated and negative control samples (stimulation index). Statistical comparisons between groups were performed, using Kruskal–Wallis and Dunn’s multiple comparisons tests.

To test immunological cross-reactivity, we performed T-cell assays with overlapping 15-mer peptides covering the two possible self-epitopes identified by blastp in SNTG1 and POMT1 (Supplementary Table 2). Interestingly, increased IFN-γ responses to peptide IFSALVVAWYSSACH (POMT1_675–689_) were demonstrated in NT1 patients (*p* < 0.01, Fig. 5a; Supplementary Fig. 5A). This suggested that POMT1 could represent an autoantigen in NT1. As seen for T-cell responses to peptide NA_175–189_, homozygosity for HLA-DQB1*0602 did not appear to augment responses to POMT1_675–689_ beyond the levels reached in HLA-DQB1*0602 heterozygous NT1 patients. In contrast, peptide RAEKTFSVYEIMCKI (SNTG1_330–344_) was recognized only by one NT1 patient, and by vaccinated controls (n.s., Fig. 5b; Supplementary Fig. 5B).

IFN-γ responses to peptide NA_175–189_ and POMT1_675–689_, or NA_178–192_ and POMT1_675–689,_ showed correlation, further suggesting cross-reactivity of specific, virus-directed T-cells with this self-epitope in NT1 (Spearman’s rank correlation coefficients *r*_s_ = 0.511 and *r*_s_ = 0.778, respectively (*p* = 0.078; **p = 0.007)).

### Transcriptomic analyses of PBMC stimulated with influenza A H1N1 NA_175–189_ or human POMT1_675–689_ indicate activation of T helper 1-cell and cytotoxic T-cell responses with cross-reactivity in Pandemrix-associated NT1

**Fig. 6:**
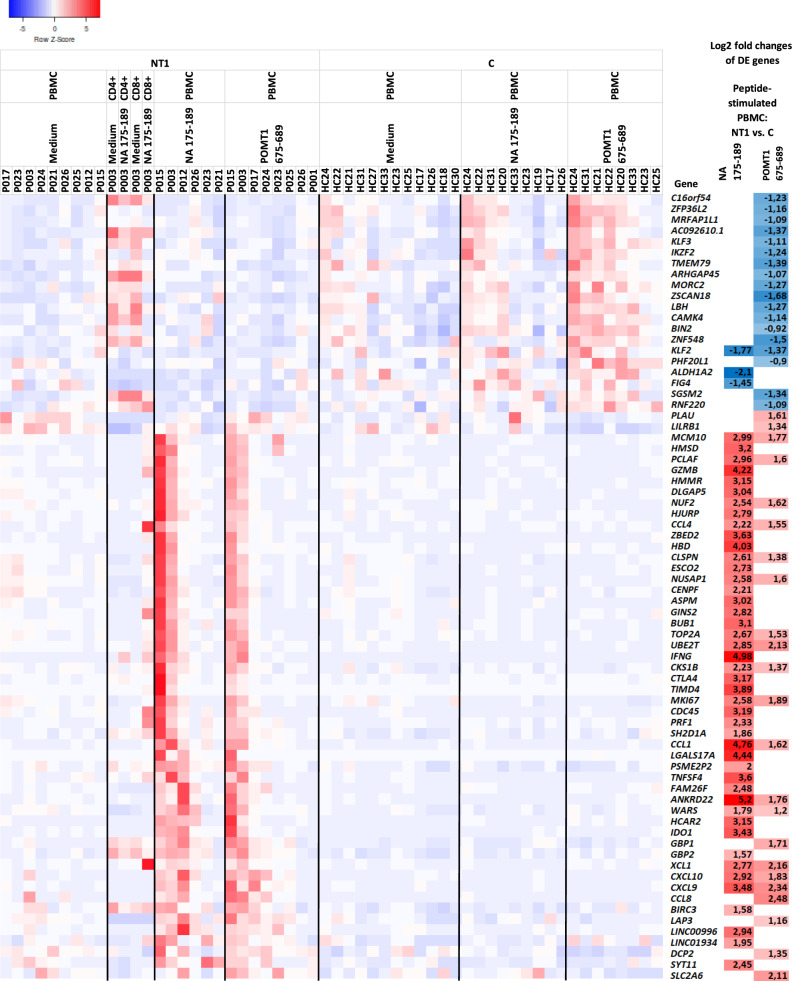
Gene expression profiling by RNA-seq of peptide-stimulated PBMC from Pandemrix-associated NT1 patients. Hierarchically clustered heatmap of the gene expression levels (*z*-score scaling generated with the Heatmapper program) in all tested PBMC samples for 74 genes showing significant differences in expression (**p < 0.01) between NT1 patients and controls either after stimulation with NA_175–189_ (49 genes) or POMT1_675–689_ peptides (28 genes; overlap of 3). Differentially expressed genes were identified using edgeR, based on a test analogous to Fisher’s exact test. The paired method was used for comparisons, calculating 2-sided *p*-values and adjusting for multiple testing using BH correction. If significance was reached (*p < 0.05), log2 fold changes between NT1 patients and controls are shown on the right. Data derived from FACS-sorted CD4+ and CD8+ cells available from one NT1 patient were added for comparison.

**Fig. 7:**
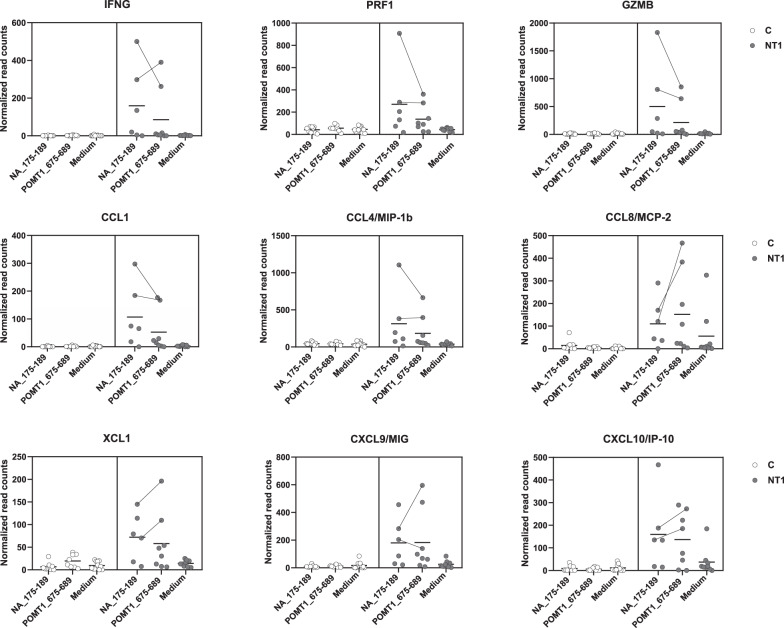
T cell-related genes differentially expressed in peptide-stimulated PBMC from Pandemrix-associated NT1 patients (RNA-seq). Dot plots demonstrating the expression levels (normalized read counts) of nine T cell-related genes showing significant differences in expression (**p < 0.01) between NT1 patients and controls either after stimulation with NA_175–189_ or POMT1_675-689_ peptides (compare Fig. 6). Differentially expressed genes were identified using edgeR, based on a test analogous to Fisher’s exact test. The paired method was used for comparisons, calculating 2-sided *p*-values and adjusting for multiple testing using BH correction. The lines indicate paired data of two strong responders, NT1 patients P003 and P015.

To further characterize T-cell responses against the epitopes from influenza A (H1N1) virus NA and human POMT1, and to identify putative pathogenic pathways in NT1, we studied the gene expression levels of viral NA_175–189_- or human POMT1_675–689_-stimulated PBMC from Pandemrix-associated NT1 patients and Pandemrix-vaccinated healthy controls. In RNA sequencing, stimulation induced a clear response in NT1 patients versus healthy controls. Altogether 74 genes were differentially expressed, either in response to NA_175–189_ (49 genes), or POMT1_675–689_ (28 genes; overlap of 3 genes, **p < 0.01; Fig. 6, Supplementary Fig. 6, Supplementary Data 2). The results further demonstrated that the gene expression profiles of PBMC from NT1 patients responding to these two peptides were closely matched (Fig. 6), and that no genes were differentially expressed in PBMC from NT1 patients stimulated with either one of the peptides (*p*-level < 0.05). These findings confirmed the correlation analyses for IFN-g responses, and supported T-cell cross-reactivity between NA and POMT1. The list of 74 differentially expressed genes contained several genes closely associated with cytotoxic T-cell function, e.g., *IFN-γ*, chemokines/chemokine ligands known to be associated with *IFN-γ*, *perforin 1*, and *granzyme B*. A focused view onto T-cell effector molecules also showed that T-cells from individual NT1 patients responded similarly to stimulation either with viral NA_175–189_ or human POMT1_675–689_, consistent with cross-reactivity of specific, influenza A (H1N1) virus-directed T-cells with the autoantigen POMT1 in NT1 (Fig. 7). This was exemplified most clearly by two strong responders, NT1 patients P003 and P015 (Figs. 6, 7), both characterized clinically by rapid onset of disease with the development of excessive daytime sleepiness and cataplexy within 4 weeks from vaccination with Pandemrix.

In addition, RNA sequencing of FACS-sorted, NA_175–189_-stimulated CD4+ and CD8+ T-cell populations from a single pediatric, Pandemrix-associated NT1 patient (P003) revealed upregulation of *IFN-γ, perforin 1, granzyme B, chemokine (C–C motif) ligand 4*, and *chemokine (C motif) ligand* in CD8+ T-cells, while the gene expression profile of CD4+ T-cells from the same patient was characterized mainly by *IFN-γ* (Fig. 6; FACS gating and sort strategy shown in Supplementary Fig. 7). Overall, gene expression in the NA_175–189_-stimulated CD8+ T-cell sample matched the profile obtained from NA_175–189_-stimulated PBMC, suggesting that peptide NA_175–189_ might be presented by both HLA class I and II. This finding was also consistent with the notion that cytotoxic T-cells are a key effector cell population mediating immunity against influenza A virus^20^.

### T-cell receptor sequencing demonstrates converging selection of influenza A H1N1 NA_175–189_- and human POMT1_675–689_-reactive clonotypes in Pandemrix-associated NT1

**Fig. 8:**
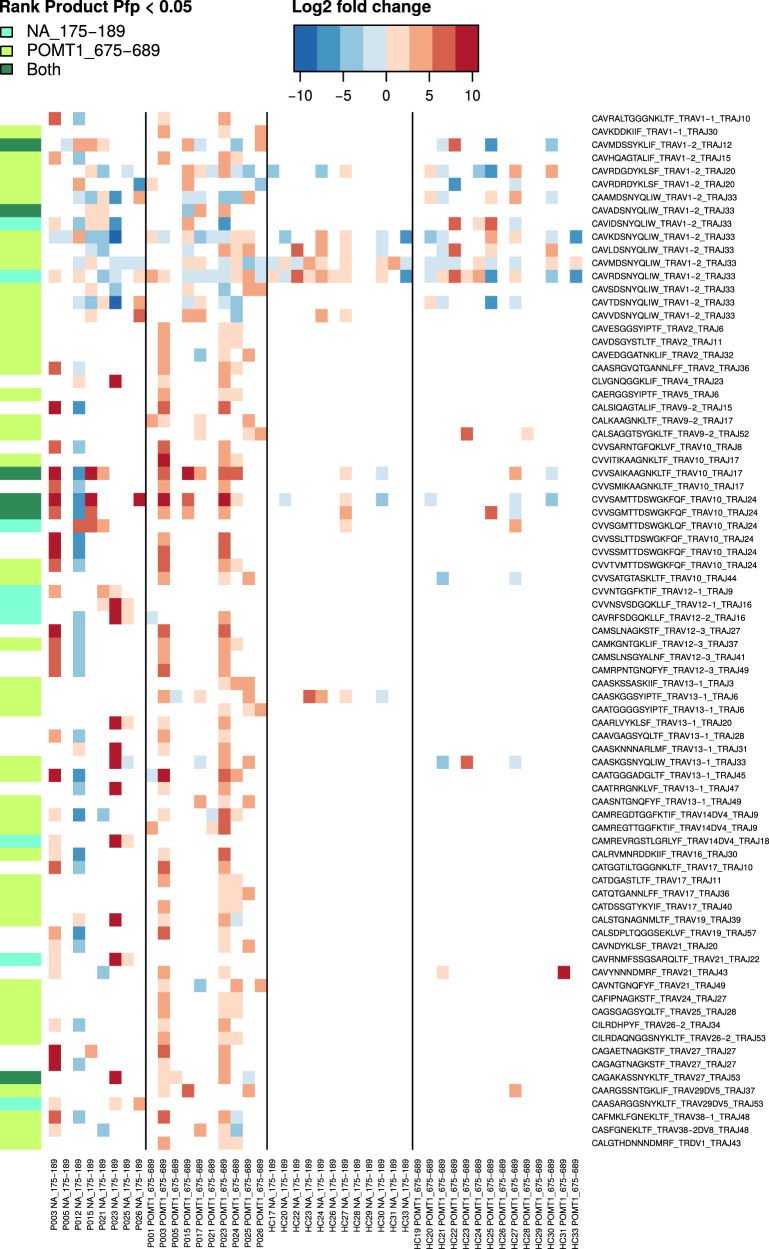
TCR α chain sequencing of peptide-stimulated PBMCs from Pandemrix-associated NT1 patients. TCR α chains (TRA) were sequenced from the same RNA samples as used for RNA sequencing. P: patient; HC: healthy control. Displayed are TRA clonotypes that were upregulated in ≥3 NT1 patient samples, based on fold changes between peptide- (NA_175–189_- or POMT1_675–689_-) treated and medium control samples from the same participant in data normalized by downsampling (heatmap). Statistical significance on the group level (NT1 patients only; Rank products test; proportion of false positives <0.05) is indicated on the left side bar.

To characterize T-cell responses against the putative cross-reactive epitopes at the clonal level, we sequenced *TCR α-chains (TRA)* and *β-chains (TRB)* from RNA of viral NA_175–189_- or human POMT1_675–689_-stimulated PBMC from Pandemrix-associated NT1 patients and Pandemrix-vaccinated healthy controls. *TRA variable (TRAV), TRA joining (TRAJ), TRB variable (TRBV), TRB diversity (TRBD)*, and *TRB joining (TRBJ)* gene segment usage did not differ significantly between peptide-stimulated and medium control samples, neither in patients nor controls (Supplementary Data 3). We identified TRA clonotypes that were upregulated in ≥3 NT1 patient samples, stimulated either with NA_175–189_ or POMT1_675–689_ peptides (Fig. 8). Among these public TRA clonotypes, six clonotypes significantly increased in abundance both when stimulated with NA_175–189_, or POMT1_675–689_ (proportion of false positives <0.05), further supporting T-cell cross-reactivity. Most strikingly, clonotypes CVV**SA**IKAAGNKLTF-TRAV10/TRAJ17 and CVV**SAM**TTDSWGK**F**QF-TRAV10/TRAJ24 were upregulated in 3 of 8 (3 of 8) NA_175–189_-stimulated, and 5 of 10 (4 of 10) POMT1_675–689_-stimulated samples. In NT1 patients P003 and P015, characterized by T helper 1 (Th1)-cell/cytotoxic T-cell gene expression signatures in response to stimulation with NA_175–189_ or POMT1_675–689_, both clonotypes were upregulated in response to either of the two peptides. Three additional clonotypes were found, significantly more abundant after stimulation with at least one of the two peptides, and with high similarity to either of the two clonotypes above: CVV**IT**IKAAGNKLTF-TRAV10/TRAJ17, significantly upregulated in POMT1_675–689_-stimulated samples, including P003; CVV**SGM**TTDSWGK**L**QF-TRAV10/TRAJ24, upregulated in NA_175–189_-stimulated samples, including P015; and CVV**SGM**TTDSWGK**F**QF-TRAV10/TRAJ24, upregulated in both NA_175–189_- and POMT1_675–689_-stimulated samples, including both P003 and P015. These results demonstrated converging selection of NA_175–189_- and/or POMT1_675–689_-reactive T-cell clones using TRAV10/TRAJ17 or TRAV10/TRAJ24 gene segments in Pandemrix-associated NT1 patients.

**Fig. 9:**
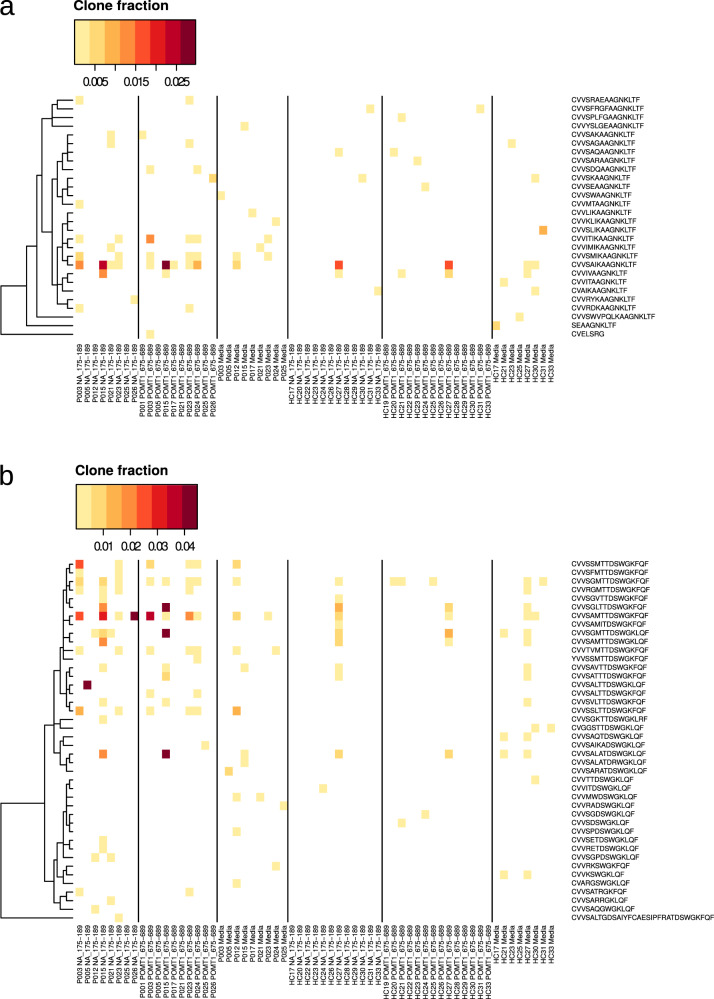
T cell clones using TRAV10–TRAJ17 and TRAV10–TRAJ24 gene segments. TCR α chains (TRA) were sequenced from the same RNA samples as used for RNA sequencing (compare Fig. 8). P: patient; HC: healthy control. Heatmaps present clone fractions, i.e. the proportions of clones using TRAV10–TRAJ17 (**a**) and TRAV10–TRAJ24 (**b**) of all clones in total data. Clones are clustered based on CDR3 sequence.

A complete list of both public and private clonotypes using TRAV10/TRAJ17 and TRAV10/TRAJ24, identified in NT1 patients or healthy controls, is provided in Fig. 9a, b. The results demonstrate that in addition to the identified public clonotypes using TRAV10/TRAJ17 (CVV**SA**IKAAGNKLTF, CVV**IT**IKAAGNKLTF) and TRAV10/TRAJ24 (CVV**SAM**TTDSWGK**F**QF, CVV**SGM**TTDSWGK**L**QF, CVV**SGM**TTDSWGK**F**QF), several highly similar clonotypes were present in peptide-stimulated samples from various NT1 patients, including CVV**SM**IKAAGNKLTF-TRAV10/TRAJ17, CVV**SSM**TTDSWGK**F**QF-TRAV10/TRAJ24, CVV**TVM**TTDSWGK**F**QF-TRAV10/TRAJ24, and CVV**SSL**TTDSWGK**F**QF-TRAV10/TRAJ24. The identified public clonotypes were not exclusive to NT1 patients, but occasionally seen in healthy controls, too. In particular, the HLA-DQB1*0602-negative healthy control HC27 shared several of the clonotypes upregulated in NT1 patient samples stimulated with NA_175-189_ or POMT1_675-689_. However, in contrast to NT1 patients, HC27 did not show an increase in expression of inflammatory cytokines/ chemokines in response to peptide stimulation (compare Fig. 4a, b; 5a; 7). Therefore, these TRAV10–TRAJ17 and TRAV10–TRAJ24 clonotypes might have contributed to an inflammatory reaction in NT1 patients, but their functional phenotype could be effectively regulated when present in healthy controls.

Applying the same search criteria as used for TRA, we also identified a smaller number of TRB clonotypes that were upregulated in ≥3 NT1 patient samples, stimulated either with NA_175–189_ or POMT1_675–689_ peptides (Supplementary Fig. 8). Among these public TRB clonotypes, again one clonotype significantly increased in abundance in both NA_175–189_- or POMT1_675–689_-stimulated patient samples, further supporting T-cell cross-reactivity. This clonotype was CASSEAGQGAYEQYF-TRBV6-1/TRBJ2–7 (upregulated in P003, P015, and P023).

### Discovery of POMT1 autoantibodies in Pandemrix-vaccinated children

**Fig. 10:**
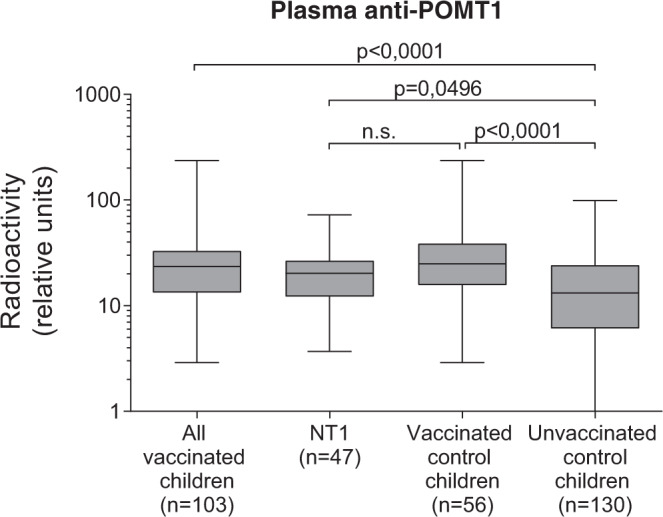
Autoantibodies in Pandemrix-associated NT1. Plasma from pediatric Pandemrix-associated NT1 patients (NT1) or pediatric Pandemrix-vaccinated or unvaccinated healthy controls were analyzed by POMT1 radioimmunoassay. Radioactivity is expressed in relative units (RU). The medians (line in box), 75 and 25% quartiles (upper and lower box boundaries) and maxima/minima (upper and lower whiskers) of each group are displayed. Statistical comparisons between groups were performed, using Kruskal–Wallis and Dunn’s multiple comparisons tests.

Finally, we studied levels of plasma antibodies to POMT1 by radioimmunoassay (RIA), a sensitive and specific liquid-phase method for the detection of autoantibodies recognizing conformational epitopes^21^. Autoantibodies to POMT1 were increased in Pandemrix-vaccinated children (both in NT1 patients and healthy controls) in comparison to unvaccinated control children, suggesting that Pandemrix induced autoimmunity to POMT1 (****p < 0.0001, Fig. 10; Table 1).

## Discussion

Genetic studies strongly implicate HLA-DQ6.2-restricted CD4+ T-cells in the pathogenesis of NT1^4,5^. A central role for CD4+ T-cells is seen in many autoimmune disorders, e.g., in celiac disease and type 1 diabetes. Both disorders show exceptionally strong HLA class II associations^22^, to be surpassed still in NT1. HLA class II-associated autoimmune diseases are considered antigen driven (e.g., by wheat gliadin in celiac disease), although tissue pathology is not mediated by CD4+ T-cells only. Other immune cells are involved in tissue destruction, and antigen spreading leads to broad autoimmunity. In Pandemrix-associated NT1, we hypothesized that CD4+ T-cells primed by influenza A (H1N1) virus epitopes are disease drivers, cross-react with CNS autoantigens, and orchestrate an immune-mediated attack against the hypocretin neuronal network. Therefore, we focused our study on influenza A (H1N1) T-cell epitopes that are recognized in the context of HLA-DQ6.2, although HLA-DQ6.2 peptide binding was not directly tested. Due to limitations in the availability of blood samples from pediatric, Pandemrix-associated NT1 patients, we started to search for dominant T-cell epitopes using Pandemrix-primed mice that expressed HLA-DQ6.2 as the only MHC class II allele, and Pandemrix-vaccinated, HLA-DQB1*0602-positive individuals. Choosing this strategy, the selected T-cell peptides were enriched for HLA-DQ6.2-binding, but likely included both MHC class II- and/or class I-binding peptides.

We found that T-cell responses against two NA- and NP-derived epitopes were enhanced in children and adolescents with Pandemrix-associated NT1 in comparison to Pandemrix-vaccinated controls. Further, our study provided evidence for influenza A (H1N1) virus-directed T- and B-cell cross-reactivity with human POMT1. Homology of a viral NA peptide sequence and a human POMT1 sequence was suggested by basic local alignment. We could show that IFN-γ secretion in response to NA_175–189_/NA_178–192_- and POMT1_675–689_-stimulation was correlated, and that T cell cytokine/chemokine gene expression patterns were closely matched. This was exemplified by two strong responders, patients P003 and P015, who both developed clinical narcolepsy with cataplexy within one month from Pandemrix vaccination. TCR repertoire analyses in NT1 patients revealed increased abundance of seven public clonotypes in response to stimulation both with NA_175–189_ and POMT1_675–689_ in NT1 patients. In addition, vaccination with Pandemrix induced anti-POMT1 autoantibodies in children. These results were consistent with cross-reactivity of specific, virus-directed T-cells and antibodies with the autoantigen POMT1 in Pandemrix-associated NT1.

The reasons remain unknown for the strongly enhanced T-cell reactivity to different influenza A (H1N1) protein fragments demonstrated in this study in patients who developed NT1 after Pandemrix vaccination. Our earlier study showed that viral proteins contained in the Pandemrix antigen suspension formed complexes, which may have contributed to the enhanced reactivity to both NA- and NP-derived epitopes^16^. Alternatively, and not mutually exclusive, enhanced T- and B-cell reactivity in NT1 patients may be attributed to immunogenetic predispositions^6–8,23^, resulting in hyper-responsiveness to pro-inflammatory signals and AS03 adjuvant-supplemented influenza A (H1N1) priming.

Specific HLA class I alleles, most consistently HLA-A*11:01, account for additional genetic risk contributions in NT1, independent from HLA-DQB1*06:02^24,25^. Cytotoxic CD8+ T cells, likely acting in concert with CD4+ Th1-cells, are frequently implicated in the pathogenesis of autoimmune diseases, including the neurological disorders multiple sclerosis^26^ and NT1^25^. In this study of Pandemrix-associated NT1, cytokine and chemokine gene expression by PBMC in response to stimulation with influenza A (H1N1) NA_175–189_ or human POMT1_675–689_ matched closely, and revealed a cytotoxic T cell signature, characterized by *IFN-γ, IFN-γ−*associated genes*, perforin 1*, and *granzyme B*. This phenotype was consistent with CD8+ T-cells, but also unconventional, cytotoxic CD4+ T-cells, previously observed in influenza A infection^20^. In FACS-sorted T cell fractions, available for study from one Pandemrix-associated NT1 patient only, *perforin 1* and *granzyme B* expression was upregulated in NA_175–189_-stimulated CD8+ T-cells, but not in CD4+ T cells. Thus, NA_175–189_ peptide is most likely binding to both HLA class I and II molecules.

Six TRA clonotypes, and one TRB clonotype, significantly increased in abundance in samples from Pandemrix-associated NT1 patients both when stimulated with NA_175–189_, or with POMT1_675–689_. Strikingly, public TRA clonotypes CVVSAIKAAGNKLTF-TRAV10/TRAJ17 and CVVSAMTTDSWGKFQF-TRAV10/TRAJ24 were upregulated in 37.5% of NA_175–189_-stimulated, and 40–50% of POMT1_675–689_-stimulated samples. In addition, we identified several public TRA clonotypes with high similarity to these two dominant clonotypes in samples from NT1 patients. These results suggest converging selection in different NT1 patients of NA_175–189_- and/or POMT1_675–689_-reactive T-cell clones using TRAV10/TRAJ17 or TRAV10/TRAJ24 gene segments. This indicates that these clonotypes are likely involved in the pathogenesis of NT1.

However, the identified public clonotypes were not exclusive to NT1 patients. Occasionally, they were seen in Pandemrix-vaccinated healthy controls, too. Interestingly, a HLA-DQB1*0602-negative healthy control shared both dominant TRAV10–TRAJ17 and TRAV10–TRAJ24 clonotypes, indicating that T-cells expressing these clonotypes were probably not HLA-DQ6.2-restricted. Therefore, TRAV10–TRAJ17 and TRAV10–TRAJ24 clonotypes might have contributed to an inflammatory reaction in NT1 patients, likely driven by CD8+ T-cells recognizing NA_175–189_ and putative cross-reactive POMT1_675–689_ (auto-) antigens, while the same clonotypes appeared effectively regulated when present in healthy controls. Use in future studies of single cell technology may allow the direct functional characterization of T-cell clonotypes in NT1 patients and controls.

Of special interest is the identification of CVVSAMTTDSWGK**F**QF-TRAV10/TRAJ24 as a dominant public clonotype, and of additional, highly similar TRAV10–TRAJ24 clonotypes, upregulated in NA_175–189_- or POMT1_675–689_-stimulated samples from NT1 patients. Han et al. described a single nucleotide polymorphism (SNP) associated with NT1, rs1483979. This SNP was located within the *TRAJ24* segment and changed an amino acid within the CDR3 peptide-binding site of the TCR (F8L)^27^. Of the top public TRAV10–TRAJ24 clonotypes detected in samples from NT1 patients in our study, two encoded for phenylalanine (F), and one for leucine (L). All TRAJ24 (and 3 of 4 TRAJ17) clonotypes that were upregulated in response to NA_175–189_- or POMT1_675–689_-stimulation shared TRAV10 gene segment usage. These clonotypes differed from TRAV2–TRAJ24^18^ and TRAV6–TRAJ24^28^ clonotypes, previously isolated from NT1 patients using HLA-DQ6.2-HCRT tetramers. To our knowledge, clonotypes using TRAV10, TRAJ17 or TRBV6–1/TRBJ2–7 have not been described in NT1 patients before. The dominant public TRAV10–TRAJ17, TRAV10–TRAJ24, and TRBV6–1/TRBJ2–7 clonotypes described here represent potential biomarkers for NT1.

Among the identified T-cell epitopes, IAYERMCNILKG (NP_217–228_; Immune Epitope Database ID 145824), derived from the NYMC X-157 vaccine strain, is shared between different influenza A virus strains. In contrast, ESVAWSASACHD (NA_175-186_; ID 97390) and MERNAGSGIIISDTP (HA_274-288_; ID 188723) are carried mainly by influenza A (H1N1) virus strains. A recent study by Luo et al. demonstrated an increased frequency of T-cells specific for HA_273–287_ in NT1 patients in comparison to HLA-DQB1*0602 positive controls^18^. However, a report by Schinkelshoek et al. found no significant increase in T cell reactivity against HA_273–287_ in NT1 patients in comparison to HLA-DQB1*0602 positive controls^29^. Peptides HA_271–285_ and HA_274–288_ were recognized by patients with NT1 and healthy vaccinees in our study cohort, confirming HA_273–287_ as an influenza A (H1N1) T-cell epitope. Yet again, we did not see a clear-cut difference between NT1 patients and healthy vaccinees, neither in comparison to HLA-DQB1*0602 positive or negative controls, questioning the role of HA_273–287_-reactivity in the pathogenesis of NT1.

Using HLA-DQ6.2 tetramers, the study by Luo et al. demonstrated the presence of CD4+ T-cells specific for C-terminally amidated, human HCRT_54–66_ and HCRT_86–97_ peptides, but not for native HCRT peptides, in NT1 patients and controls^18^. Furthermore, TCR sequencing analyses suggested cross-reactivity with HA. The recent study by Jiang et al., also using HLA-DQ6.2 tetramers, detected CD4+ T cells specific for native HCRT_56–69_ and HCRT_87–100_ peptides in NT1 patients and controls^28^. In addition, Latorre et al. reported increased frequencies of predominantly HLA-DR-restricted CD4+ T cell clones recognizing much more diverse peptide epitopes of HCRT in NT1 patients. These clones did not show reactivity with HA. Notably, T-cell reactivity to HCRT was also detected in type 2 narcolepsy, which is not associated with HCRT-deficiency^30^. Thus, the current literature on T cell reactivity to HCRT in NT1 remains conflicting. In many autoimmune diseases, autoimmunity is directed against multiple autoantigens expressed in the target tissue. The results of our study do not exclude a role of autoimmunity to HCRT, but identify with POMT1 another autoantigen in NT1.

POMT1 is a glycosyltransferase anchored in the endoplasmic reticulum, catalyzing in complex with POMT2 the first step of O-mannosyl glycan synthesis. In humans, O-mannose-linked glycosylation is observed mainly in brain, peripheral nerve, and muscle glycoproteins. The best-known substrate of POMT1/POMT2 is α-dystroglycan, mediating the binding of extracellular matrix components such as laminin to the dystrophin complex. Congenital defects in POMT1 are a cause of muscular dystrophy-dystroglycanopathy types A1, B1, and C1^31^. Some forms of muscular dystrophies, most clearly myotonic dystrophy type 1, are associated with rapid eye movement (REM) sleep dysregulation^32^. It is currently unknown whether POMT1 or its substrates are involved in neuronal sleep regulation, but this possibility could be explored in experimental studies.

In this study, T-cell reactivity with POMT1 was detected in Pandemrix-associated NT1 patients. Samples from unvaccinated NT1 patients were not available for comparisons. Currently, it remains unknown whether T cell reactivity against POMT1 can be observed in NT1 patients not exposed to influenza A (H1N1) vaccination or infection. Other questions to be addressed by future studies include the identities of the HLA alleles involved in the restriction of the dominant public TRAV10–TRAJ17, TRAV10–TRAJ24, and TRBV6–1/TRBJ2–7 clonotypes described, their function, antigenic peptide specificity and cross-reactivity, and their value for NT1 diagnosis.

In summary, this study provides evidence for an influenza A (H1N1) virus directed, POMT1 cross-reactive T-cell response in NT1, in the context of HLA-DQ6.2. We identified two dominant T-cell epitopes of influenza A (H1N1) NA and (NYMC X-157 vaccine strain) NP virus proteins in Pandemrix-associated NT1 patients. Patients mounted a vigorous Th1-cell/cytotoxic T-cell response against these epitopes and showed T-cell reactivity against a self-epitope in POMT1, an enzyme expressed in brain. The POMT1 epitope is a peptide mimic of a dominant T-cell epitope of influenza A (H1N1) NA in Pandemrix-associated NT1. *IFN-γ, perforin 1, granzyme B,* and chemokine gene expression in response to stimulation with T-cell peptides NA_175–189_ or POMT1_675–689_ was closely matched in individual NT1 patients. TCR repertoire analyses demonstrated converging selection of TRAV10/TRAJ17 and TRAV10/TRAJ24 clonotypes in NT1, upregulated in response to either NA_175–189_- or POMT1_675–689_-stimulation, thus supporting T-cell cross-reactivity and a pathogenic role of these clonotypes in NT1. Moreover, vaccinees had elevated antibody levels against human POMT1, suggesting that Pandemrix vaccination triggered an autoimmune response to POMT1. The results of this unbiased search identify POMT1 as an autoantigen recognized by T and B cells in NT1.

## Methods

### Vaccine

The 2-component vaccine Pandemrix, containing inactivated influenza A/California/7/2009 (H1N1) split virus (3.75 μg per 0.5 ml emulsion), and AS03 adjuvant (10.69 mg squalene, 11.86 mg DL-alpha-tocopherol and 4.86 mg polysorbate 80 per 0.5 ml emulsion), was obtained from GlaxoSmithKline Biologicals, Rixensart, Belgium.

### Mice

Homozygous HLA-DQA1*0102, -DQB1*0602, human CD4-transgenic Ab0 NOD mice (“HLA-DQ6.2 mice”; stock number 006023) were purchased from Jackson Laboratories, Bar Harbor, ME, USA, and bred at the University of Helsinki Laboratory Animal Centre^17^. All mice were housed under specific pathogen-free conditions, under a 14–10 h light–dark cycle, at 22 ± 2 °C and 50–60% humidity. Pandemrix vaccine was injected under isoflurane anesthesia into the right thigh muscle (50 μl emulsion volume; male or female mice; 3 months of age), followed by one booster injection after 14 days. After 6–9 weeks from the first injection, mice were exsanguinated by retro-orbital bleeding under ketamine/xylazine anesthesia (Intervet International, Boxmeer, The Netherlands; Bayer HealthCare, Leverkusen, Germany), and spleens were harvested for collection of cells.

### Peptides and recombinant proteins

15-mer peptides covering hemagglutinin (GenBank entry ACP41953.1) and neuraminidase (YP_009118627.1) of influenza (A/California/07/2009 (H1N1)), and nucleoprotein (ADE29096.1) of influenza (A/reassortant/NYMC X-179A (California/07/2009 × NYMC X-157)(H1N1)) with 12 amino acid overlap were produced. 2 × 5 15-mer peptides with 12 amino acid overlap, covering 2 viral peptide mimotopes identified in POMT1 (AAH65268.1) and SNTG1 (AAI04830.1), respectively, were also produced (all peptides from New England Peptides, Gardner, MA, USA). Peptides that failed in-house quality control by mass spectrometry were excluded (0–5.4% of viral peptides). Peptides were dissolved in endotoxin-free water/DMSO (50%; both from Sigma-Aldrich, Schnelldorf, Germany), and stored as 25 mM stock solutions at −70 °C, until the day of use. Recombinant influenza A H1N1 (A/California/07/2009) hemagglutinin (cat#11085-V08H), (A/California/04/2009) neuraminidase (cat#11058-VNAHC or cat#11058-V01H), and (A/California/07/2009) or (A/Puerto Rico/8/34/Mount Sinai) nucleoprotein (cat#40205-V08B and cat#11675-V08B) were purchased from Sino Biological, Beijing, China. Endotoxin-free, recombinant human protein-O-mannosyltransferase 1 and syntrophin gamma 1 were from Origene, Rockville, Maryland, and endotoxin-free ovalbumin from Hyglos, Bernried, Germany.

### Mouse spleen cell stimulation

Spleen cells from 1 to 2 individual mice were pooled, and seeded in triplicates in 96-well plates at 2 × 10^5^ cells/well in RPMI 1640 medium (200 μl volume) containing heat-inactivated fetal calf serum (10%), penicillin/streptomycin, glutamine, and HEPES (25 mM), at 37 °C and 5% CO_2_ (all from Sigma-Aldrich). Cells were stimulated for 6 days with 15-mer peptides (10 μM; resulting in DMSO concentration 0.1%), a combination of anti-CD3 and anti-CD28 antibodies (positive control, 3 μg and 2 μg/ml; clones 145–2C11 and 37.51; eBioscience, San Diego, CA), endotoxin-free ovalbumin (negative control), recombinant influenza A hemagglutinin, neuraminidase or nucleoprotein (all 10 μg/ml). For each stimulation, two plates were run in parallel (from two different spleen cell pools).

### Patient and healthy control donor material

The T cell study included 28 pediatric Pandemrix-associated NT1 patients (20 in the discovery cohort, and 14 in the validation cohort; 6 patients were included in both cohorts), and 33 healthy Pandemrix-vaccinated control children or adolescents (Table 1, Supplementary Table 3A, B). If results from different experiments were pooled for analysis, and two results from a single patient were available, only the result obtained with the sample drawn earlier was used. PBMC from Pandemrix-vaccinated HLA-DQB1*0602-positive individuals (sleep clinic patients without a diagnosis of NT1) were also used for influenza A (H1N1) virus T cell epitope screening (Supplementary Table 1). The plasma study included 37 pediatric Pandemrix-associated NT1 patients, 57 healthy Pandemrix-vaccinated control children or adolescents, and 130 healthy control children or adolescents that had not received Pandemrix vaccine (Table 1). NT1 patients were diagnosed in outpatient clinics at Finnish university hospitals, by pediatric neurologists, pediatricians or neurologists with expertise in sleep medicine. All NT1 patients were diagnosed based on criteria defined in the third edition of the International Classification of Sleep Disorders^33^. All NT1 patients suffered from excessive daytime sleepiness. They also had abnormal Multiple Sleep Latency Tests in polysomnography (sleep latency <8 min and ≥2 sleep onset REM periods), and either cataplexy, or cerebrospinal fluid HCRT 1 levels below 110 pg/ml, as measured using the standardized Phoenix RIA method with Stanford reference.

### Human plasma and peripheral blood mononuclear cell (PBMC) separation and storage

Whole blood was drawn and heparinized. Plasma was separated from fresh heparinized blood samples, and stored at −70 °C, before isolation of PBMC by Ficoll (GE Healthcare, Uppsala, Sweden) isogradient centrifugation. PBMC were stored in liquid nitrogen until further analysis.

### Genotyping of the HLA-DQB1*0602 allele

Genotyping for the HLA-DQB1 locus was performed using a homogeneous assay based on asymmetrical PCR and hybridization with sequence-specific lanthanide labeled oligonucleotide probes, as described earlier^34^. In short, blood dried on sample collection cards was used for asymmetric PCR where the sequence of interest was simultaneously amplified and detected using lanthanide labeled probes with locked nucleic acid additions. Complementary quencher oligonucleotides were also included in the reactions. Two different specific probes, one labeled with europium and another with terbium, were present in each reaction and allowed simultaneous use of two different probes and their individual detection by two signals measured after the amplification. Presence of DQB1*0602 allele was deduced based on a positive signal with DQB1*0602/ *0603 specific probe and a negative with DQB1*0603/*0604 specific probe. All oligonucleotides used in this study are defined in Supplementary Table 4.

### Human PBMC stimulation

Frozen PBMCs were thawed and allowed to recover in RPMI 1640 culture medium (Life Technologies, Paisley, United Kingdom) containing 2 mmol L-glutamine, 25 mmol/l HEPES, 25 μg/ml gentamycin (Sigma-Aldrich) and 10% of heat-inactivated AB serum (Innovative Research, Novi, MI, USA), for 1 h in an incubator at 37 °C and 5% CO_2_. Cells were seeded in duplicates or triplicates in 96-well round-bottom plates (Nunc, Roskilde, Denmark) at 2 × 10^5^ cells/well, using RPMI 1640 culture medium supplemented with 5% of heat-inactivated AB serum, 2 mmol L-glutamine, 25 mmol/lHEPES and 25 μg/ml gentamycin (200 µl/well), and stimulated for 6 days with 15-mer peptides (10 μM; resulting DMSO concentration 0.1%), tetanus toxoid (20 μg/ml), PBS (negative control), recombinant influenza A hemagglutinin, neuraminidase or nucleoprotein (all at 10 μg/ml). Generally, samples were stimulated with different T cell peptides selected at random. Peptide testing was prioritized, and proteins were only tested if enough cells were available. For epitope mapping, peptides were not selected at random. Instead, more samples were used for testing the inner three than the outer two peptide epitopes. No patient or control samples that had passed viability control (trypan blue staining, positive response to stimulation with tetanus control) were excluded from the study.

### FACS sorting

To study gene expression in CD4+ and CD8+ T cells, PBMCs were stimulated for 6 days with 15-mer peptides, as described in the previous section. Stimulated cells were stained with anti-CD3-Efluor 610 (BioLegend), anti-CD4-FITC (BD Pharmingen), anti-CD8-AF700 (eBioscience) and SYTOX^®^ Blue (Thermo Fisher Scientific). A BD Influx flow cytometer (BD Biosciences) was used to sort CD4+ and CD8+ T cells. Dead cells and monocytes were excluded from analysis by appropriate gating strategies and SYTOX^®^ Blue staining (gating and sort strategy shown in Supplementary Fig. 7).

### Mouse cytokine RT-qPCR analysis

Total RNA from stimulated murine cells was isolated with the RNeasy Mini kit (Qiagen, Hilden, Germany), and cDNA was synthesized using a high-capacity cDNA reverse transcription kit (Applied Biosystems, Foster City, CA, USA). TaqMan gene expression assays (*IFN-γ* Mm01168134 _m1; Rn18S Mm03928990_g1), TaqMan® Fast Universal PCR Master Mix 10 × 250 Rxn and a StepOne Plus instrument (all Applied Biosystems) were used for real-time detection of *IFN-γ* target gene complementary DNA amplification. *Ribosomal 18S* was used as endogenous reference. Relative expression was calculated by the 2^−ΔΔCt^ method.

### Cytokine fluorescent multiplex bead-based immunoassay

The cytokines IFN-γ and IL-2 were measured in cell culture supernatants using human or mouse Milliplex MAP Kits (HCYTMAG-60K, MCYTMAG-70K, Millipore, Billerica, MA, USA), according to the manufacturer’s instructions. Quantification was performed with a Magpix instrument and xPONENT 4.2 or 4.3 software (Luminex Corp., Austin, TX), or Bio-Plex 200® System and Bio-Plex Manager software version 5.0 (BIO-RAD Laboratories, Hercules, CA, USA). The concentration of each cytokine was determined from an 8-point standard curve using five parameter logistic regression. The samples below minimum detectable concentration (MinDC) were given an arbitrary value of 50% of MinDC. Each sample was compared to an unstimulated sample.

### Basic local alignment of protein sequences

Basic local alignment of protein sequences was performed using the web-based BLASTP program search tool (version 2.2.32+) at the National Centre for Biotechnology Information (NCBI; https://blast.ncbi.nlm.nih.gov/).

### RNA extraction from human PBMC and sorted CD4+ and CD8+ T-cells

RNA was prepared from stimulated PBMC stored at −70 °C in RLT buffer (Qiagen), using the RNeasy Mini Kit (Qiagen, Hilden, Germany) and following the manufacturer’s instructions. Cells were homogenized with QIAshredder columns (Qiagen). After extraction, RNA was quantified using a NanoDrop spectrophotometer (Thermo Fisher Scientific). RNA samples were stored at −70 °C. RNA was captured from stimulated CD4+ or CD8+ T cells stored at −20 °C in RNAlater (Thermo Fisher Scientific) after FACS sorting, according to a published protocol^35^. Briefly, samples were mixed with lysis buffer (0.3% (v/v) Triton X100, 20 mM DTT, 2 mM dNTPs) in ratio 1:1 and vortexed. Magnetic Dynabeads (M-270 Streptavidin, Thermo Fisher Scientific) coated with RNA capturing polyT-tailed Indexing Oligonucleotides (Integrated DNA Technologies) were added to each well. After 5 min of incubation the magnetic beads were separated from the supernatant and washed twice with 6X SSC buffer. Subsequently, the beads were combined with RT mix as described later.

### RNA Sequencing

The in-house 3′ bulk RNA-sequencing “3B-seq” method was modified from the single cell Drop-seq protocol^36^, as described^37^. Briefly, 10 ng of RNA was mixed with indexing oligonucleotides (Integrated DNA Technologies, Coralville, IA) and after 5 min of incubation, the RNA was combined with RT mix (1× Maxima RT buffer, 1 mmol/L deoxynucleoside triphosphate, 10 U/μL Maxima H-RTase (all Thermo Fisher Scientific), 1 U/μL RNase inhibitor (Lucigen, Middleton, WI), and 2.5 μmol/L Template Switch Oligo (TSO; Integrated DNA Technologies, Coralville, IA)). Primers are listed in Supplementary Table 4. Samples were incubated in a T100 thermal cycler (Bio-Rad) for 30 min at 22 °C and 90 min at 52 °C. The constructed complementary DNA (cDNA) was amplified by PCR using the RT mix as template and adding 1× HiFi HotStart Readymix (Kapa Biosystems, Wilmington, MA), and 0.8 μmol/L SMART PCR primer. The samples were thermocycled in a T100 thermocycler (Bio-Rad) as follows: 95 °C for 3 min; then 4 cycles of 98 °C for 20 s, 65 °C for 45 s, 72 °C for 3 min; then 16 cycles of 98 °C for 20 s, 67 °C for 20 s, 72 °C for 3 min; and with the final extension step of 5 min at 72 °C. An aliquot of this PCR product was used for TCR sequencing (below). The PCR products were pooled together in sets of 12 samples containing different indexing oligos and purified with 0.6× HighPrep PCR reagent (MAGBIO, Gaithersburg, USA) according to the manufacturer’s instructions. They were eluted in 10 μL of molecular grade water. The 3′-end complementary DNA fragments for sequencing were prepared using the Nextera XT (Illumina) tagmentation reaction. The reaction was performed according to the manufacturer’s instructions, except for the use of P5 SMART primer, which replaced the S5xx Nextera primer. Each set of 12 samples that was pooled after the PCR reaction was tagmented with a different Nextera N7xx index. Subsequently, the samples were PCR amplified as follows: 95 °C for 30 seconds, 11 cycles of 95 °C for 10 seconds, 55 °C for 30 seconds, and 72 °C for 30 seconds, with the final extension step of 5 minutes at 72 °C. Samples were purified twice using 0.6× and 1.0× HighPrep PCR reagent and eluted in 10 μL of molecular-grade water. The concentration of the libraries was measured by using a Qubit 2 fluorometer (Invitrogen) and the Qubit DNA HS Assay Kit (Thermo Fisher Scientific). The quality of the sequencing libraries was assessed using the LabChip GXII Touch HT electrophoresis system (PerkinElmer), with the DNA High Sensitivity Assay (PerkinElmer) and the DNA 5K/RNA/Charge Variant Assay LabChip (PerkinElmer). Samples were stored at −20 °C. The libraries were sequenced on an Illumina NextSeq 500 instrument, with a custom primer (DS Custom Read 1) producing read 1 of 20 base pairs (bp), and read 2 (paired end) of 50 bp. Sequencing was performed at the Functional Genomics Unit of the University of Helsinki, Finland.

### Read alignment and generation of digital expression data

Raw sequence data were processed as described before^37^. The pipeline originally suggested by Macosko et al. for processing of Drop-seq data was used^36^. Briefly, reads were filtered to remove polyA tails of length 6 or greater, and then aligned to the human (GRCh38) genome using STAR aligner v2.7.2^38^, with default settings. Uniquely mapped reads were grouped according to the 1–12 barcode, and gene transcripts were counted by their unique molecular identifiers (UMIs) to reduce the bias emerging from the PCR amplification. Digital expression matrices reported the number of transcripts per gene in each sample (according to the distinct unique molecular identifiers sequences counted). Differentially expressed genes were identified using edgeR v3.4.0, based on a test analogous to Fisher’s exact test^39^. The paired method was used for comparisons, calculating 2-sided *p*-values and adjusting for multiple testing using BH correction. RNAseq heatmaps were hierarchically clustered and drawn using the web-based Heatmapper software (accessed 25th of July 2020)^40^.

### TCR sequencing

Primers were designed for human TCR constant genes using Primer3^41,42^, and appended to a tailing sequence to produce the reverse primers N7tail-TRAC-2 and N7tail-TRBC-2 (Supplementary Table 4). Forward primers (DS_N5xx_5TSO) contained a sequence complementary to the Template Switch Oligo and an index sequence (I5). TCR sequences were amplified using KAPA HiFi HotStart Readymix, 0.3 μM of each primer and 1 μl pre-amplified cDNA library (see RNA Sequencing) in 10 μl volume with 16 cycles of 95 °C for 20 s, 62 °C for 20 s and 72 °C for 30 s, followed by purification with 1.5× volume of HighPrep PCR reagent. The TCR libraries were dual indexed using the same forward primer as in the previous step, and one of Nextera N7xx primers. PCR was done using KAPA HiFi HotStart Readymix, 0.3 μM of each primer and 3 μl template in 10 μl volume with 10 cycles of 95 °C for 20 s, 62 °C for 20 seconds and 72 °C for 30 s. The libraries were pooled according to concentration and purified first with 1× and then with 1.5× volume of HighPrep PCR reagent. The quality of the libraries was assessed with a LabChip GXII Touch HT electrophoresis instrument and quantitative PCR. Libraries were sequenced with a MiSeq Reagent Kit v3 (600-cycle) using the 300 + 300 bp paired-end protocol, a custom read 1 primer (DS-CustomRead1_5TSO) and 10% PhiX on an Illumina Miseq instrument at Department of Virology, University of Helsinki, Finland.

### TCR analysis

TCR sequencing reads were mapped and clonotypes assembled with MiXCR v3.0.12^43,44^. The NA_175–189_-stimulated sample from HC19 did not produce any clonotypes and was excluded. Low-count clones that had an identical CDR3 nucleotide sequence to a high-count clone (over 500-fold difference) were filtered out as probable cross-sample contaminants. Non-productive clones were also filtered out. To normalize the data, counts were downsampled pairwise using VDJtools v1.2.1^45^ so that each NA_175–189_-treated and medium-treated sample from the same patient was downsampled to the same total count. The same was done separately for the POMT1_675–689_-treated samples. Further processing was done using R statistical software v3.6.3 (R Foundation for Statistical Computing, Vienna, Austria). Gene usage differences between peptide and media-treated patient samples were examined using the Immunarch package v0.5.5 (Zenodo/CERN, Geneva, Switzerland) and Wilcoxon test with the paired method. *P* values were corrected for multiple testing with the Benjamini–Hochberg method. For clone-level analysis, low-count clones were filtered out by requiring each clone to have count >10 in at least one sample. One pseudocount was added, the counts were log2 transformed and fold change between the peptide-treated sample and medium control from the same individual was calculated for public clones (present in at least three samples). Statistical significance was tested with Rank Products test implemented in the RankProd package v3.12.0^46,47^, using the paired method. Clones were hierarchically clustered based on the Levenshtein edit distance of their CDR3 sequences.

### Plasma POMT1 antibody radioimmunoassay

The full-length human POMT1 cDNA (GenBank entry BC065268.1) was cloned into the pTNT vector (GenScript, Piscataway, NJ, USA) under the control of the SP6 promoter, and multiplied in Invitrogen subcloning efficiency DH5α competent cells (ThermoFisher, Waltham, MA, USA), according to the manufacturer’s instructions. ^35^S-methionine-labeled POMT1 was produced by in vitro transcription and translation of the purified plasmids using the TNT Coupled Reticulocyte Lysate System according to the manufacturer’s instructions (Promega, Madison, WI, USA) in the presence of ^35^S-methionine (Perkin Elmer, Waltham, MA, USA). Unincorporated ^35^S-methionine was removed by gel chromatography on NAP-5 columns (GE Healthcare, Chicago, IL, USA). Plasma samples were analyzed for POMT1 (auto)-antibodies by RIA, as described for GAD autoantibodies^21^. Briefly, 2 µl of plasma was incubated overnight at 4 °C in 96-well plates with 20,000 cpm of ^35^S-methionine-labeled POMT1, diluted in 50 µl of TBST buffer (50 mM Tris, 150 mM NaCl, 0.1% Tween-20, pH 7.4). Antibody–antigen complexes were precipitated with protein A-Sepharose, and unbound label was removed by washing several times with TBST buffer (50 mM Tris, 150 mM NaCl, 0.1% Tween-20, pH 7.4). The bound radioactivity was measured with a liquid scintillation counter (1450 MicroBeta Trilux, Perkin Elmer Life Sciences, Turku, Finland), and the results were expressed in relative units (RU), based on a serial dilution in-house standard curve.

### Statistics

Statistical comparisons between groups were performed, using Prism version 6.05 (Graph Pad Software, San Diego, CA), and Kruskal–Wallis and Dunn’s multiple comparisons tests. Correlation analyses were performed by calculating Spearman’s rank correlation coefficient. Additional details on the statistics used in the analysis of RNA and TCR sequencing data are included in the sections above.

### Study approval

NT1 patients were Finnish participants in the NARPANORD narcolepsy study. Heathy control children and adolescents were recruited via the Finnish Diabetes Registry. Pandemrix-vaccinated HLA-DQB1*0602-positive individuals without a diagnosis of NT1 were recruited at the Helsinki Sleep Clinic, Vitalmed Research Center, Helsinki, Finland. All participants or their guardians had given written, informed consent. Ethical permissions for clinical studies were obtained from the Ethics Committee of the Hospital District of Helsinki and Uusimaa, Finland. All animal procedures were approved by the Board for Animal Research (ELLA), Southern Finnish State Administrative Agency (ESAVI/1064/04.10.03/2012).

### Reporting summary

Further information on research design is available in the Nature Research Reporting Summary linked to this article.

## Supplementary information

Supplementary Information

Description of Additional Supplementary Files

Supplementary Data 1

Supplementary Data 2

Supplementary Data 3

Reporting Summary

## Data Availability

The sequencing data have been deposited to the EMBL-EBI European Genome-phenome Archive (EGA) under the accession number EGAS00001004886 (RNA sequencing data, Fig. 6; TCR sequencing data, Fig. 7). Upon reasonable request and subject to a material transfer agreement, data submitted to EGA will be made available by the corresponding data access committee. Source data are provided with this paper.

## Source data

Source data
