# Supplementary material for: Enhanced influenza A H1N1 T cell epitope recognition and cross-reactivity to protein-O-mannosyltransferase 1 in Pandemrix-associated narcolepsy type 1
Source: Nat Commun. 2021 Apr 16;12:2283. doi: 10.1038/s41467-021-22637-8 (PMC8052463; doi:10.1038/s41467-021-22637-8)
Supplement: Supplementary file 1 — Supplementary Information [file 41467_2021_22637_MOESM1_ESM.pdf]

Supplementary Information

Enhanced influenza A H1N1 T cell epitope recognition and cross-reactivity to protein-O-mannosyltransferase 1 in Pandemrix-associated narcolepsy type 1

A. Vuorela [1,*], T.L. Freitag [2,3,*,#], K. Leskinen [3], H. Pessa [3], T. Härkönen [1], I. Stracenski [2], T. Kirjavainen [4], P. Olsen [5], O. Saarenpää-Heikkilä [6], J. Ilonen[7,8], M. Knip [1,4,9], A. Vaheri [10], M. Partinen [1,11,12], P. Saavalainen [3], S. Meri [2,3] and O. Vaarala [1]

[1] Clinicum, University of Helsinki, Helsinki, Finland; [2] Department of Bacteriology and Immunology, [3] Translational Immunology Research Program, University of Helsinki, Helsinki, Finland; [4] Children's Hospital, University of Helsinki, and Helsinki University Hospital, Helsinki, Finland; [5] Department of Child Neurology, Oulu University Hospital, Oulu, Finland; [6] Department of Pediatrics, Tampere University Hospital, Tampere Finland; [7] Immunogenetics Laboratory, Institute of Biomedicine, University of Turku, Turku, Finland; [8] Clinical Microbiology, Turku University Hospital, Turku, Finland; [9] Research Program for Clinical and Molecular Metabolism, University of Helsinki, Helsinki, Finland; [10] Department of Virology, [11] Department of Neurosciences, University of Helsinki, Helsinki, Finland; [12] Helsinki Sleep Clinic, Vitalmed Research Center, Helsinki, Finland

* These authors contributed equally to the work

# Corresponding author

First authors:

Arja Vuorela, MSc

Tobias L. Freitag, MD

Address Correspondence to:

Tobias L. Freitag, MD
Department of Bacteriology and Immunology
Translational Immunology Research Program
Haartmaninkatu 3 (P.O. Box 21)
00014 University of Helsinki
Finland
eMail: tobias.freitag@helsinki.fi

Influenza A (H1N1) virus T-cell epitope screen in HLA-DQ6.2 mice

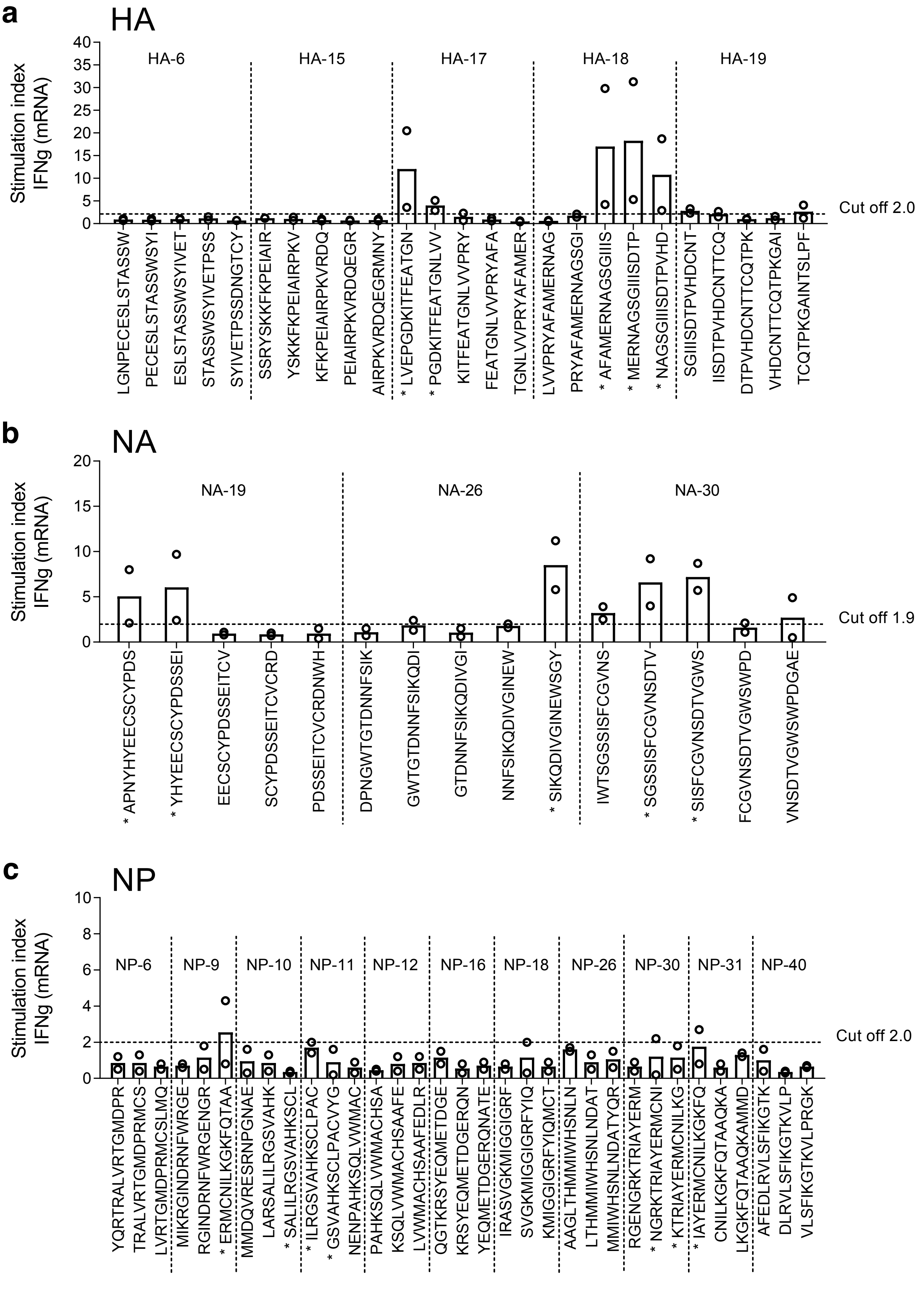

Supplementary Figure 1: Influenza A (H1N1) virus T-cell epitope screen in HLA-DQ6.2 mice. Mouse spleen cells (pooling cells from 2 x 2 mice, n=2) were stimulated in culture with single 15-mer peptides from influenza (A/reassortant/NYMC X-179A (California/07/2009 x NYMC X-157)(H1N1)) vaccine virus **A)** hemagglutinin (HA), **B)** neuraminidase (NA) or **C)** nucleoprotein (NP). The expression of *IFN-*γ was measured by RT-qPCR (mRNA). Results are expressed as ratios between relative gene expressions measured in peptide-stimulated and negative control samples (stimulation index; dots representing single values, bars representing means). An asterisk (*) indicates a single peptide that was selected for further testing.

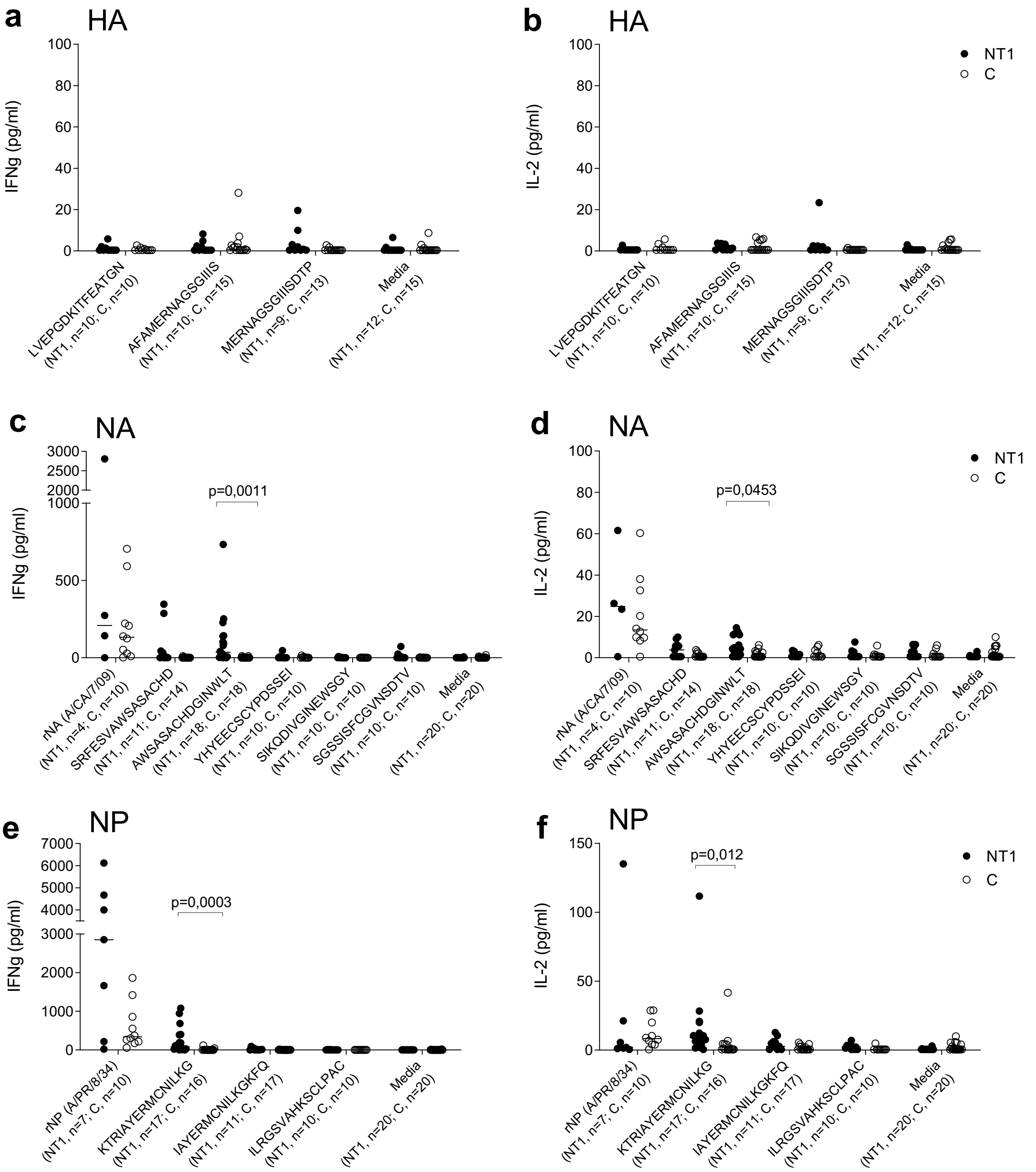

a
HA
IFNg (pg/ml)
LVEPGDKITFEATGN (NT1, n=10; C, n=10)
AFAMERNAGSGIIIS (NT1, n=10; C, n=15)
MERNAGSGIIISDTP (NT1, n=9; C, n=13)
Media (NT1, n=12; C, n=15)
b
HA
IL-2 (pg/ml)
NT1
C
c
NA
p=0,0011
rNA (A/CA/7/09) (NT1, n=4; C, n=10)
SRFESVAWSASACHD (NT1, n=11; C, n=14)
AWSASACHDGINWLT (NT1, n=18; C, n=18)
YHYEECSCYPDSSEI (NT1, n=10; C, n=10)
SIKQDIVGINEWSGY (NT1, n=10; C, n=10)
SGSSISFCGVNSDTV (NT1, n=10; C, n=10)
Media (NT1, n=20; C, n=20)
d
NA
p=0,0453
e
NP
p=0,0003
rNP (A/PR/8/34) (NT1, n=7; C, n=10)
KTRIAYERMCNILKG (NT1, n=17; C, n=16)
IAYERMCNILKGKFQ (NT1, n=11; C, n=17)
ILRGSVAHKSCLPAC (NT1, n=10; C, n=10)
Media (NT1, n=20; C, n=20)
f
NP
p=0,012

Supplementary Figure 2: Identification of influenza A (H1N1) virus T-cell epitopes in Pandemrix-associated NT1 patients. PBMC from pediatric Pandemrix-associated NT1 patients (NT1; discovery cohort) or pediatric Pandemrix-vaccinated healthy controls (C) were stimulated in culture with single 15-mer peptides from influenza (A/reassortant/NYMC X-179A (California/07/2009 x NYMC X-157)(H1N1)) vaccine virus hemagglutinin (HA), neuraminidase (NA) or nucleoprotein (NP). The secretion of IFN-γ (**A, C, E)** or IL-2 (**B, D, F)** was measured by FMIA (protein). Results are expressed as cytokine concentrations in peptide-stimulated and negative control samples (pg/ml). Statistical comparisons between groups were performed, using Kruskal-Wallis and Dunn's multiple comparisons tests.

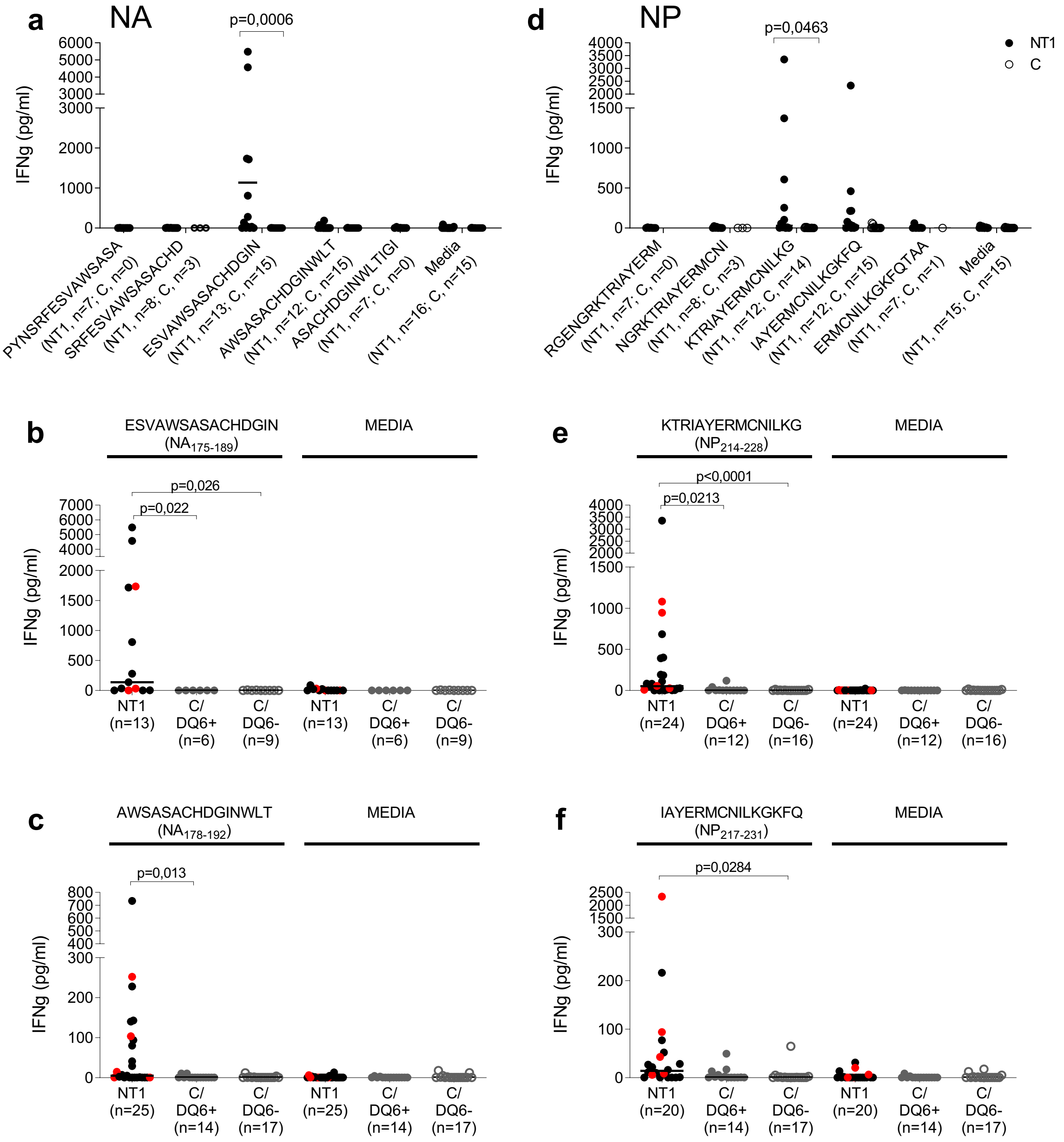
a
NA
p=0,0006
IFNg (pg/ml)
6000
5000
4000
3000
3000
2000
1000
0
PYNSRFESVAWSASA
(NT1, n=7; C, n=0)
SRFESVAWSASACHD
(NT1, n=8; C, n=3)
ESVAWSASACHDGIN
(NT1, n=13; C, n=15)
AWSASACHDGINWLT
(NT1, n=12; C, n=15)
ASACHDGINWLTIGI
(NT1, n=7; C, n=0)
Media
(NT1, n=16; C, n=15)
d
NP
p=0,0463
NT1
C
IFNg (pg/ml)
4000
3500
3000
2500
2000
1500
1000
500
0
RGENGRKTRIAYERM
(NT1, n=7; C, n=0)
NGRKTRIAYERMCNI
(NT1, n=8; C, n=3)
KTRIAYERMCNILKG
(NT1, n=12; C, n=14)
IAYERMCNILKGKFQ
(NT1, n=12; C, n=15)
ERMCNILKGKFQTAA
(NT1, n=7; C, n=1)
Media
(NT1, n=15; C, n=15)
b
ESVAWSASACHDGIN
(NA175-189)
MEDIA
p=0,026
p=0,022
IFNg (pg/ml)
7000
6000
5000
4000
2000
1500
1000
500
0
NT1 (n=13)
C/ DQ6+ (n=6)
C/ DQ6- (n=9)
NT1 (n=13)
C/ DQ6+ (n=6)
C/ DQ6- (n=9)
e
KTRIAYERMCNILKG
(NP214-228)
MEDIA
p<0,0001
p=0,0213
IFNg (pg/ml)
4000
3500
3000
2500
2000
1500
1000
500
0
NT1 (n=24)
C/ DQ6+ (n=12)
C/ DQ6- (n=16)
NT1 (n=24)
C/ DQ6+ (n=12)
C/ DQ6- (n=16)
c
AWSASACHDGINWLT
(NA178-192)
MEDIA
p=0,013
IFNg (pg/ml)
800
700
600
500
400
300
200
100
0
NT1 (n=25)
C/ DQ6+ (n=14)
C/ DQ6- (n=17)
NT1 (n=25)
C/ DQ6+ (n=14)
C/ DQ6- (n=17)
f
IAYERMCNILKGKFQ
(NP217-231)
MEDIA
p=0,0284
IFNg (pg/ml)
2500
2000
1500
300
200
100
0
NT1 (n=20)
C/ DQ6+ (n=14)
C/ DQ6- (n=17)
NT1 (n=20)
C/ DQ6+ (n=14)
C/ DQ6- (n=17)

Supplementary Figure 3: Mapping of identified influenza A (H1N1) virus neuraminidase and nucleoprotein T-cell epitopes in Pandemrix-associated NT1 patients. A), D) PBMC from pediatric Pandemrix-associated NT1 patients (NT1; validation cohort) or pediatric Pandemrix-vaccinated healthy controls (C) were stimulated in culture with overlapping 15-mer peptides from influenza (A/reassortant/NYMC X-179A (California/07/2009 x NYMC X-157)(H1N1)) vaccine virus neuraminidase (NA) or nucleoprotein (NP), as indicated. **B), C), E), F)** PBMC from NT1 patients (invariably *HLA-DQB1*0602* positive; discovery and validation cohorts combined; *HLA-DQB1*0602* homozygous NT1 patients marked with red dots) and *HLA-DQB1*0602* positive (C/DQ6+) or negative (C/DQ6-) healthy controls were stimulated with single NA- or NP-derived peptides. The secretion of IFN-γ was measured by FMIA (protein). Results are expressed as cytokine concentrations in peptide-stimulated and negative control samples (pg/ml). Statistical comparisons between groups were performed, using Kruskal-Wallis and Dunn’s multiple comparisons tests.

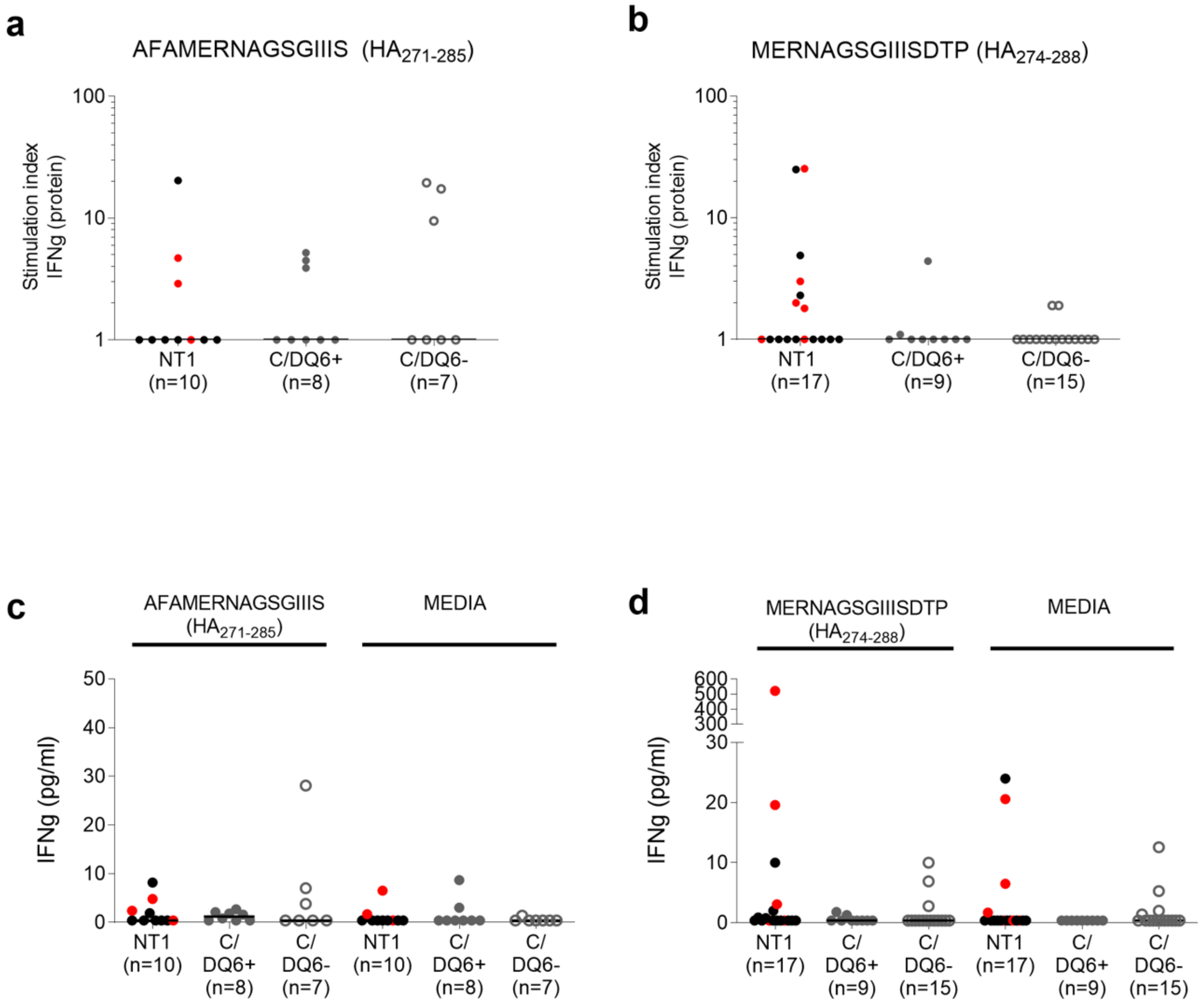
a
AFAMERNAGSGIIIS (HA271-285)
Stimulation index IFNg (protein)
100
10
1
NT1 (n=10)
C/DQ6+ (n=8)
C/DQ6- (n=7)
b
MERNAGSGIIISDTP (HA274-288)
Stimulation index IFNg (protein)
100
10
1
NT1 (n=17)
C/DQ6+ (n=9)
C/DQ6- (n=15)
c
AFAMERNAGSGIIIS (HA271-285)
MEDIA
IFNg (pg/ml)
50
40
30
20
10
0
NT1 (n=10)
C/ DQ6+ (n=8)
C/ DQ6- (n=7)
NT1 (n=10)
C/ DQ6+ (n=8)
C/ DQ6- (n=7)
d
MERNAGSGIIISDTP (HA274-288)
MEDIA
IFNg (pg/ml)
600
500
400
300
30
20
10
0
NT1 (n=17)
C/ DQ6+ (n=9)
C/ DQ6- (n=15)
NT1 (n=17)
C/ DQ6+ (n=9)
C/ DQ6- (n=15)

Supplementary Figure 4: Influenza A (H1N1) virus hemagglutinin T-cell epitopes in Pandemrix-associated NT1 patients. PBMC from NT1 patients (invariably *HLADQB1*0602* positive in this cohort; discovery and validation cohorts combined; *HLA-DQB1*0602* homozygous NT1 patients marked with red dots) and *HLA-DQB1*0602* positive (C/DQ6+) or negative (C/DQ6-) healthy controls were stimulated with single hemagglutinin (HA)-derived peptides. The secretion of IFN-γ was measured by FMIA (protein). Results are expressed as the ratio between cytokine concentrations measured in peptide-stimulated and negative control samples (stimulation index; **A, B)**, or as cytokine concentrations in single samples (pg/ml; **C, D)**. Statistical comparisons between groups were performed, using Kruskal-Wallis and Dunn's multiple comparisons tests.

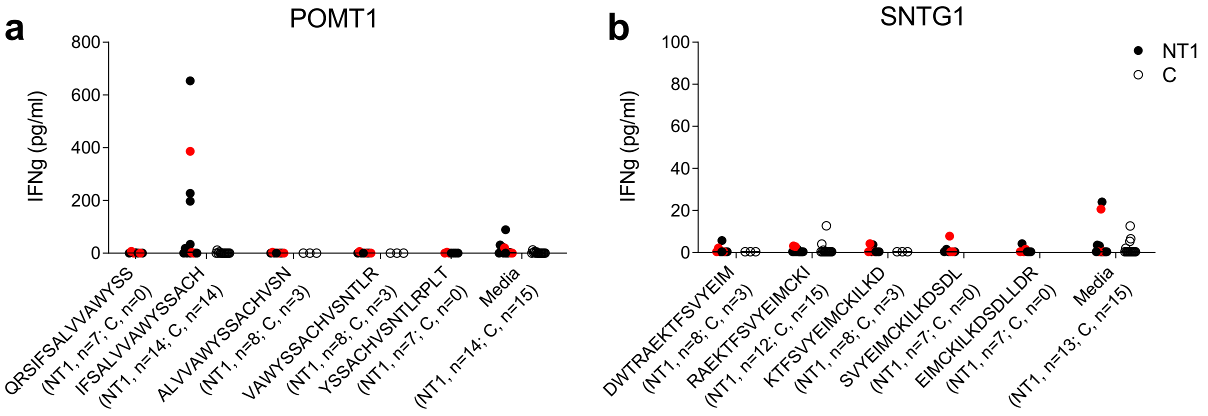

Supplementary Figure 5: Autoreactive T-cells in Pandemrix-associated NT1. A), B) PBMC from pediatric Pandemrix-associated NT1 patients (NT1) or pediatric Pandemrix-vaccinated healthy controls (C) were stimulated in culture with overlapping 15-mer peptides derived from human protein-O-mannosyl transferase 1 (POMT1) or syntrophin gamma-1 (SNTG1), as indicated. The secretion of IFN-γ was measured by FMIA (protein). *HLA-DQB1*0602* homozygous NT1 patients are marked with red dots. Results are expressed as cytokine concentrations measured in peptide-stimulated and negative control samples (pg/ml). Statistical comparisons between groups were performed, using Kruskal-Wallis and Dunn’s multiple comparisons tests.

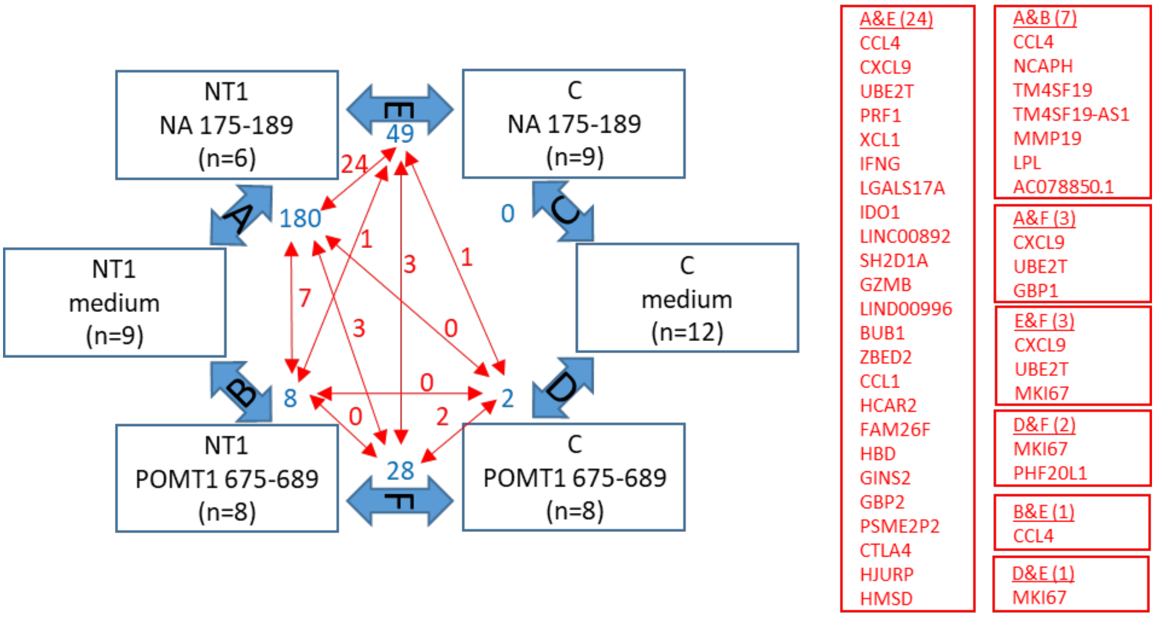

Supplementary Figure 6: RNA sequencing study design; number and identity of differentially expressed genes. Differentially expressed (DE-) genes in group comparisons A-F (see below) were identified using edgeR, based on a test analogous to Fisher's exact test. The paired method was used for comparisons, calculating 2-sided p-values and adjusting for multiple testing using BH correction. A full list of DE-genes is provided under Supplementary Data 2. The numbers of DE-genes significant on a cut-off level of **p (adjusted) <0.01 are indicated in blue. The numbers of overlapping genes between the DE lists A-F are indicated in red. These genes are also listed in the red boxes. The numbers of patient or control samples passing quality check and included in analysis are provided. DE-genes from comparisons E and F are also listed in the heatmap in Figure 6 (cut-off level of p (adjusted) <0.01). **A:** NT1 patient PBMC, stimulated with $NA_{175-189}$ peptide vs. medium. **B:** NT1 patient PBMC, stimulated with $POMT1_{675-689}$ peptide vs. medium. **C:** Healthy control PBMC, stimulated with $NA_{175-189}$ peptide vs. medium. **D:** Healthy control PBMC, stimulated with $POMT1_{675-689}$ peptide vs. medium. **E:** NT1 patient vs. healthy control PBMC, both stimulated with $NA_{175-189}$ peptide. **F:** NT1 patient vs. healthy control PBMC, both stimulated with $POMT1_{675-689}$ peptide.

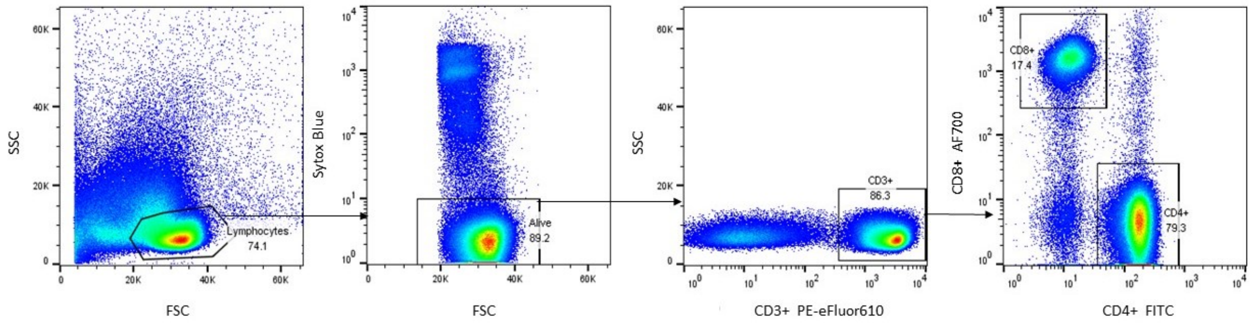

Supplementary Figure 7: FACS gating and sort strategy used for the isolation of CD4+ and CD8+ T cells from NA$_{175\text{-}189}$-stimulated (or medium control) PBMC samples from NT1 patient P003. Stimulated cells were stained with anti-CD3-Efluor 610, anti-CD4-FITC, anti-CD8-AF700 and SYTOX® Blue (dead cell dye). Lymphocytes were selected and dead cells were excluded from analysis as shown. Subsequently, CD3+ T cells were selected, and CD4 or CD8 single positive cells were sorted for further analysis.

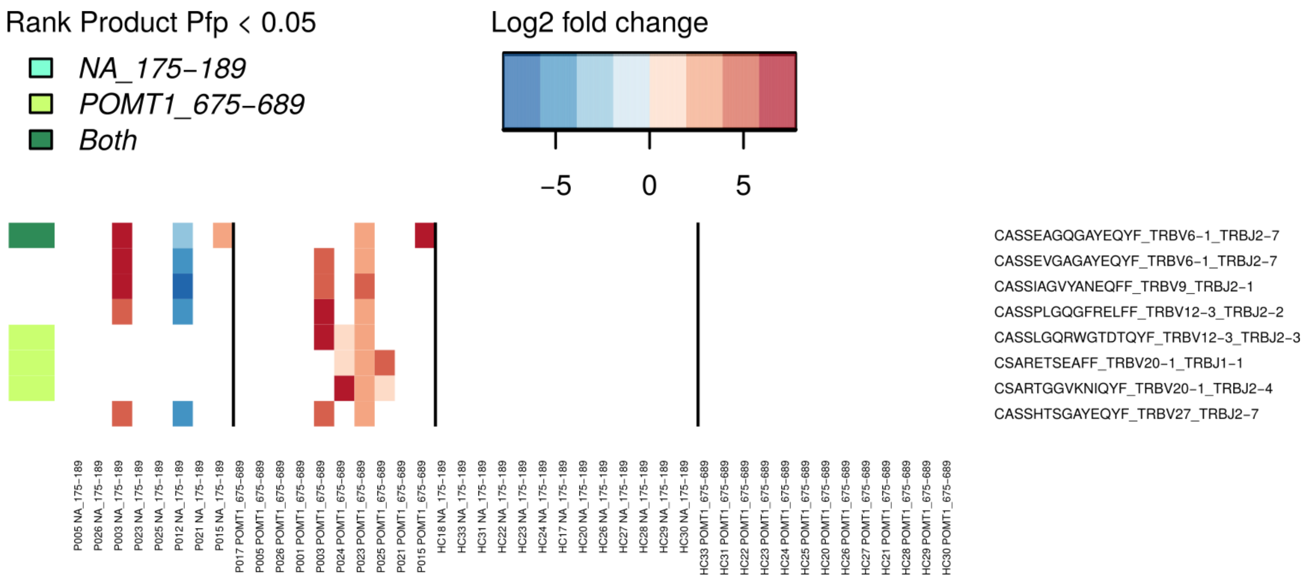

Supplementary Figure 8: *TCR β chain* sequencing of peptide-stimulated PBMCs from Pandemrix-associated NT1 patients. *TCR β chains (TRB)* were sequenced from the same RNA samples as used for RNA sequencing. P: patient; HC: healthy control. Displayed are TRB clonotypes that were upregulated in ≥3 NT1 patient samples, based on fold changes between peptide- (NA$_{175-189}$- or POMT1$_{675-689}$-) treated and medium control samples from the same individual in data normalized by down sampling (heatmap). Statistical significance on the group level (NT1 patients only; Rank products test; proportion of false positives <0.05) is indicated on the left side bar.

ID	Gender (0=male, 1=female)	DQB1*0602 (0=no, 1= yes)	Pandemrix vaccination (0=no, 1= yes)	Age at vaccination (years)	Age at sampling (years)	Vaccination to sampling (d)
PRO-001	1	1	1	15,0	18,2	1174
PRO-002	0	1	1	not available	19,3	not available
PRO-003	1	1	1	14,6	17,8	1156

Supplementary Table 1: List of Pandemrix-vaccinated, *HLA-DQB1*0602* positive sleep clinic patients without a diagnosis of NT1, whose PBMC samples were used in experiments, and clinical information.

Discovery/ Validation	Source protein	Sequence	Position; Comment
	**HA**	LVEPGDKITFEATGN	HA 250-264
	**HA**	AFAMERNAGSGIIIS	HA 271-285
	**HA**	MERNAGSGIIISDTP	HA 274-288; Luo G et al. [18]
	**NA**	SRFESVAWSASACHD	NA 172-186
	**NA**	AWSASACHDGINWLT	NA 178-192
	**NA**	YHYEECSCYPDSSEI	NA 274-288
	**NA**	SIKQDIVGINEWSGY	NA 388-402
	**NA**	SGSSISFCGVNSDTV	NA 439-453
	**NP**	KTRIAYERMCNILKG	NP 214-228
	**NP**	IAYERMCNILKGKFQ	NP 217-231
	**NP**	ILRGSVAHKSCLPAC	NP 265-279
**Mapping**	**NA**	PYNSRFESVAWSASA	NA 169-183
	**NA**	SRFESVAWSASACHD	NA 172-186
	**NA**	ESVAWSASACHDGIN	NA 175-189
	**NA**	AWSASACHDGINWLT	NA 178-192
	**NA**	ASACHDGINWLTIGI	NA 181-195
	**NP**	RGENGRKTRIAYERM	NP 208-222
	**NP**	NGRKTRIAYERMCNI	NP 211-225
	**NP**	KTRIAYERMCNILKG	NP 214-228
	**NP**	IAYERMCNILKGKFQ	NP 217-231
	**NP**	ERMCNILKGKFQTAA	NP 220-234
	**POMT1**	QRSIFSALVVAWYSS	POMT1 672-686
	**POMT1**	IFSALVVAWYSSACH	POMT1 675-689
	**POMT1**	ALVVAWYSSACHVSN	POMT1 678-692
	**POMT1**	VAWYSSACHVSNTLR	POMT1 681-695
	**POMT1**	YSSACHVSNTLRPLT	POMT1 684-698
	**SNTG1**	DWTRAEKTFSVYEIM	SNTG1 327-341
	**SNTG1**	RAEKTFSVYEIMCKI	SNTG1 330-344
	**SNTG1**	KTFSVYEIMCKILKD	SNTG1 333-347
	**SNTG1**	SVYEIMCKILKDSDL	SNTG1 336-350
	**SNTG1**	EIMCKILKDSDLLDR	SNTG1 339-353

Supplementary Table 2: List of 15-mer peptides, used for stimulation of PBMC from Pandemrix-associated NT1 patients or control.

Supplementary table 3A

ID	No	Gender (0=male, 1=female)	DQB1*0602 (0=no, 1= yes)	Pandemrix vaccination (0=no, 1= yes)	Age at vaccination	Age at sampling	Vaccination to sampling (d)	Vaccination to onset (d)	Experiment
P001	1	0	1	1	16,6	17,7	413	32	D
P001	2	0	1	1	16,6	21,1	1640	32	V
P002	1	0	1	1	13,8	15,1	456	83	D
P003	1	1	1	1	10,8	12,3	538	19	D
P003	2	1	1	1	10,8	15,2	1626	19	V
P004	1	0	1	1	7,7	9,2	551	28	D
P004	2	0	1	1	7,7	10,6	1089	28	D
P005	1	1	1	1	14,9	16,4	546	197	D
P005	2	1	1	1	14,9	17,9	1075	197	V
P005	3	1	1	1	14,9	19,7	1763	197	V
P006	1	1	1	1	8,0	9,6	567	37	D
P007	1	0	1	1	11,8	13,4	561	62	D
P008	1	1	1	1	11,1	12,7	591	29	D
P009	1	1	1	1	6,6	8,0	531	186	D
P010	1	0	1	1	13,4	15,2	629	262	D
P011	1	0	1	1	20,9	23,1	805	101	D
P012	1	0	1	1	6,9	9,2	849	24	D
P012	2	0	1	1	6,9	11,8	1781	24	V
P013	1	1	1	1	13,3	15,9	915	89	D
P014	1	1	1	1	5,1	7,7	939	76	D
P015	1	1	1	1	10,7	13,4	1004	8	D
P015	2	1	1	1	10,7	15,4	1728	8	V
P016	1	1	1	1	8,1	11,1	1079	75	D
P017	1	0	1	1	11,1	14,0	1078	50	D,V
P018	1	1	1	1	13,8	16,8	1077	96	D
P019	1	1	1	1	8,3	11,3	1085	29	D
P020	1	1	1	1	11,8	15,1	1201	0	V
P021	1	0	1	1	14,7	19,5	1718	93	V
P022	1	1	1	1	12,9	16,2	1214	35	D
P023	1	0	1	1	15,5	20,0	1645	133	V
P024	1	1	1	1	16,1	20,6	1645	11	V
P025	1	1	1	1	18,6	23,1	1645	13	V
P026	1	1	1	1	11,0	14,2	1168	331	V
P027	1	1	1	1	7,4	10,9	1288	13	V
P028	1	1	1	1	8,8	12,7	1412	19	V

Supplementary table 3B

ID	Gender (0=male, 1=female)	DQB1*0602 (0=no, 1= yes)	Pandemrix vaccination (0=no, 1= yes)	Age at vaccination	Age at sampling	Vaccination to sampling (d)
HC01	0	0	1	11,0	12,3	476
HC02	0	0	1	10,1	11,4	455
HC03	0	0	1	5,2	6,7	527
HC04	0	0	1	4,5	6,2	599
HC05	0	1	1	4,1	5,5	516
HC06	0	1	1	10,6	13,2	946
HC07	1	0	1	16,4	18,7	835
HC08	1	1	1	7,0	8,5	562
HC09	1	0	1	9,8	11,3	566
HC10	0	0	1	8,8	10,0	441
HC11	0	0	1	7,4	8,7	480
HC12	1	0	1	6,7	7,9	434
HC13	1	1	1	6,1	6,7	208
HC14	1	1	1	13,2	14,5	475
HC15	1	1	1	4,5	6,0	550
HC16	0	1	1	6,8	7,1	122
HC17	0	0	1	6,9	8,4	532
HC18	1	1	1	10,7	10,8	13
HC19	1	1	1	5,1	5,6	197
HC20	0	0	1	13,6	15,1	534
HC21	0	0	1	13,1	14,7	575
HC22	0	1	1	10,2	10,8	205
HC23	0	1	1	8,0	8,7	247
HC24	1	0	1	8,5	9,8	476
HC25	1	0	1	5,3	6,9	566
HC26	1	0	1	10,6	12,1	544
HC27	1	0	1	13,1	14,6	531
HC28	1	0	1	5,2	6,9	600
HC29	1	0	1	14,8	16,5	591
HC30	1	0	1	7,5	9,1	610
HC31	1	1	1	7,5	7,8	108
HC32	1	1	1	9,0	10,6	581
HC33	1	1	1	14,4	14,6	88

Supplementary Table 3: A) List of NT1 patients, whose PBMC samples were used in experiments, and clinical information. D: Sample included in discovery cohort. V: Sample included in validation cohort. **B)** List of healthy individuals, whose PBMC samples were used in experiments, and clinical information.

Oligonucleotide	Probe sequence	Quencher sequence
**DQB1 probes**		
*02	AAG AGA TCG TG	CGC ACG ATC TCT
*03:01	TGG AGG TGT AC	CGG TAC ACC TCC
*03:01/3	GCC GCC TGA CG	CGT CAG GCG G
*03:02	GCC GCC TGC CG	GGC AGG CGG
*04/5	TGC GGG GTG TGA C	GTC ACA CCC CGC A
*04	AAC GGG ACC GAG C	GTC ACA CCC CGC A
*05/6	GGG CGG CCT	AGG CCG CCC
*05:01	ACC GGG CAG TGA	TCA CTG CCC GGT
*06:02/3	TAC CGC GCG	CGC GCG GTA
*06:03/4	TTG TAA CCA GAC AC	GTG TCT GGT TAC A
control	CGC TTC GAC AG	CTG TCG AAG CG
**DQB1 primers**		
5' primer	GGGCATGTGCTACTTCACCAACG	
3' primer	CCTTCTGGCTGTTCCAGTACT	
**RNAseq primers**		
TSO	AAGCAGTGGTATCAACGCAGAGTGAATrGrGrG	
SMART PCR primer	AAGCAGTGGTATCAACGCAGAGT	
P5 SMART primer	AATGATACGGCGACCACCGAGATCTACACGCCTGTCCGCGGAA GCAGTGGTATCAACGCAGAGT*A*C	
DS custom read 1	GCCTGTCCGCGGAAGCAGTGGTATCAACGCAGAGTAC	
**TCR primers**		
N7tail-TRAC-2	GTCTCGTGGGCTCGGAGATGTGTATAAGAGACAGGTCTC TCAGCTGGTACACGG	
N7tail-TRBC-2	GTCTCGTGGGCTCGGAGATGTGTATAAGAGACAGTGCTT CTGATGGCTCAAACAC	
DS_N5xx_5TSO	AATGATACGGCGACCACCGAGATCTACAC(i5)GCCTGTCCG CGGAAGCAGTGGTATCAACGCAGAGTGAAT	
Nextera_N7xx	CAAGCAGAAGACGGCATACGAGAT(i7)GTCTCGTGGGCTCGG	
DS custom read 1 5TSO	GCCTGTCCGCGGAAGCAGTGGTATCAACGCAGAGTGAAT	

Supplementary Table 4: List of oligonucleotides used for HLA-DQB1 genotyping, RNA sequencing and TCR sequencing. Underlined bases are locked nucleic acids.
