# Supplementary material for: Enhanced influenza A H1N1 T cell epitope recognition and cross-reactivity to protein-O-mannosyltransferase 1 in Pandemrix-associated narcolepsy type 1
Source: Nat Commun. 2021 Apr 16;12:2283. doi: 10.1038/s41467-021-22637-8 (PMC8052463; doi:10.1038/s41467-021-22637-8)
Supplement: Supplementary file 2 — Description of Additional Supplementary Files [file 41467_2021_22637_MOESM2_ESM.pdf]

Description of Additional Supplementary Files

Supplementary Data 1: List, and grouping into pools, of 15-mer peptides used for stimulation of T cells, from hemagglutinin of influenza (A/California/07/2009 (H1N1)), neuraminidase of influenza (A/California/07/2009 (H1N1)), and nucleoprotein of influenza (A/reassortant/NYMC X-179A (California/07/2009 x NYMC X-157)(H1N1)).

Supplementary Data 2: Lists of differentially expressed (DE-) genes in RNA sequencing. (DE-) genes were identified using edgeR, based on a test analogous to Fisher's exact test. The paired method was used for comparisons, calculating 2-sided p-values and adjusting for multiple testing using BH correction. The RNA sequencing study design is shown in Supplementary Figure 6. DE-genes identified in group comparisons A-F are listed, based on a cut-off level of *p (adjusted) <0.05. DE-genes significant on a cut-off level of **p (adjusted) <0.01 are shown in bold. DE-genes from comparisons E and F are also listed in the heatmap in Figure 6A (cut-off level of p (adjusted) <0.01).

A. NT1 patient PBMC, stimulated with $NA_{175-189}$ peptide vs. medium

B. NT1 patient PBMC, stimulated with $POMT1_{675-689}$ peptide vs. medium

C. Healthy control PBMC, stimulated with $NA_{175-189}$ peptide vs. medium

D. Healthy control PBMC, stimulated with $POMT1_{675-689}$ peptide vs. medium

E. NT1 patient vs. healthy control PBMC, both stimulated with $NA_{175-189}$ peptide

F. NT1 patient vs. healthy control PBMC, both stimulated with $POMT1_{675-689}$ peptide

Supplementary Data 3: Analysis of TRA gene segment usage in patients and controls. Gene segment usage was compared between peptide (NA_175-189 or POMT1_675-689)-stimulated and medium control samples, using Wilcoxon test with the paired method. P values were corrected for multiple testing with the Benjamini-Hochberg (BH) method.
