# Supplementary material for: Enhanced influenza A H1N1 T cell epitope recognition and cross-reactivity to protein-O-mannosyltransferase 1 in Pandemrix-associated narcolepsy type 1
Source: Nat Commun. 2021 Apr 16;12:2283. doi: 10.1038/s41467-021-22637-8 (PMC8052463; doi:10.1038/s41467-021-22637-8)
Supplement: Supplementary file 6 — Reporting Summary [file 41467_2021_22637_MOESM6_ESM.pdf]

nature research

Corresponding author(s): Tobias Freitag

Last updated by author(s): 17.3.2021

Reporting Summary

Nature Research wishes to improve the reproducibility of the work that we publish. This form provides structure for consistency and transparency in reporting. For further information on Nature Research policies, see our Editorial Policies and the Editorial Policy Checklist.

Statistics

For all statistical analyses, confirm that the following items are present in the figure legend, table legend, main text, or Methods section.

n/a	Confirmed	
☐	☒	The exact sample size (*n*) for each experimental group/condition, given as a discrete number and unit of measurement
☐	☒	A statement on whether measurements were taken from distinct samples or whether the same sample was measured repeatedly
☐	☒	The statistical test(s) used AND whether they are one- or two-sided<br>*Only common tests should be described solely by name; describe more complex techniques in the Methods section.*
☐	☒	A description of all covariates tested
☐	☒	A description of any assumptions or corrections, such as tests of normality and adjustment for multiple comparisons
☐	☒	A full description of the statistical parameters including central tendency (e.g. means) or other basic estimates (e.g. regression coefficient) AND variation (e.g. standard deviation) or associated estimates of uncertainty (e.g. confidence intervals)
☐	☒	For null hypothesis testing, the test statistic (e.g. *F*, *t*, *r*) with confidence intervals, effect sizes, degrees of freedom and *P* value noted<br>*Give P values as exact values whenever suitable.*
☒	☐	For Bayesian analysis, information on the choice of priors and Markov chain Monte Carlo settings
☒	☐	For hierarchical and complex designs, identification of the appropriate level for tests and full reporting of outcomes
☐	☒	Estimates of effect sizes (e.g. Cohen's *d*, Pearson's *r*), indicating how they were calculated

Our web collection on statistics for biologists contains articles on many of the points above.

Software and code

Policy information about availability of computer code

Data collection	1) In RNA sequencing: Sequence reads were filtered and aligned using Star aligner software v2.7.2, with default settings.<br>2) In TCR sequencing: TCR sequencing reads were mapped and clonotypes assembled with MiXCR v3.0.12 software.
Data analysis	1) In RNA sequencing: Differentially expressed genes were identified using edgeR software v3.4.0. Heatmaps were drawn using the web-based Heatmapper software (accessed 25th of July 2020).<br>2) In TCR sequencing: Details of the analysis are provided in the EMBL-EBI European Genome-phenome Archive (EGA) submission. Clonotype data was downsampled pairwise using VDJtools v1.2.1 software. Further data processing was done using R statistical software v3.6.3 (R Foundation for Statistical Computing, Vienna, Austria). Gene usage was examined using the Immunarch package v0.5.5 (Zenodo/CERN, Geneva, Switzerland). Statistical significance was tested using the RankProd package v3.12.0.

For manuscripts utilizing custom algorithms or software that are central to the research but not yet described in published literature, software must be made available to editors and reviewers. We strongly encourage code deposition in a community repository (e.g. GitHub). See the Nature Research guidelines for submitting code & software for further information.

Data

Policy information about availability of data

All manuscripts must include a data availability statement. This statement should provide the following information, where applicable:

- Accession codes, unique identifiers, or web links for publicly available datasets
- A list of figures that have associated raw data
- A description of any restrictions on data availability

The raw data underlying the results depicted in Figures 2, 3, 4, 5 and 10, and in Supplementary Figures 1, 2, 3, 4 and 5, is provided as a source data file. The sequencing data has been deposited to the EMBL-EBI European Genome-phenome Archive (EGA) under the accession number EGAS00001004886 (RNA sequencing

data, Figure 6; TCR sequencing data, Figure 7). Upon reasonable request and subject to a material transfer agreement, data submitted to EGA will be made available by the corresponding data access committee.

Field-specific reporting

Please select the one below that is the best fit for your research. If you are not sure, read the appropriate sections before making your selection.

☒ Life sciences ☐ Behavioural & social sciences ☐ Ecological, evolutionary & environmental sciences

For a reference copy of the document with all sections, see nature.com/documents/nr-reporting-summary-flat.pdf

Life sciences study design

All studies must disclose on these points even when the disclosure is negative.

Sample size	Sample size was based on previous experience with similar studies in patients. All samples available were used. Reference example for sample size in a PBMC study: Savilahti EM et al. Clin Immunol 136:16-20, 2010. Reference example for sample size in plasma antibody study: Savola K et al. Diabetologia 41:1293-97, 1998.
Data exclusions	No data was excluded from patients or controls after having passed inclusion criteria (e.g. fullfilling diagnostic criteria), provided that the data passed pre-established technical quality control criteria. PBMC samples were excluded if trypan blue staining indicated insufficient cell viability. In sequencing data, some samples did not produce reads, and were excluded on this basis.
Replication	The T cell study was designed using patient discovery and validation cohorts. Overall design is depicted in Figure 1, and allowed for verification of positive results at every successive step. Only verified positive results were considered further. PBMC assays were performed using triplicates. RNA sequencing and TCR sequencing was performed once, due to the high cost involved. The plasma antibody study (POMT1 RIA) was performed twice, and the results were confirmed. These assays were done using duplicates.
Randomization	Randomization was not relevant for the study. Mouse cells used for stimulations were pooled from individual mice. Patient and control groups were defined based on clinical diagnostic criteria.
Blinding	This experimental study was not blinded, because the readout was not observer- or patient-dependent.

Reporting for specific materials, systems and methods

We require information from authors about some types of materials, experimental systems and methods used in many studies. Here, indicate whether each material, system or method listed is relevant to your study. If you are not sure if a list item applies to your research, read the appropriate section before selecting a response.

Materials & experimental systems

n/a	Involved in the study	
☐	☒	Antibodies
☒	☐	Eukaryotic cell lines
☒	☐	Palaeontology and archaeology
☐	☒	Animals and other organisms
☐	☒	Human research participants
☒	☐	Clinical data
☒	☐	Dual use research of concern

Methods

n/a	Involved in the study	
☒	☐	ChIP-seq
☒	☐	Flow cytometry
☒	☐	MRI-based neuroimaging

Antibodies

Antibodies used	Purified anti-mouse CD3epsilon IgG, from hamster, clone 145-2C11; purified anti-mouse CD28 IgG, from hamster, clone 37.51. Both from Biolegend, USA
Validation	Both antibodies were purified by affinity chromatography, and are specified low endotoxin and azide-free, for use in functional cellular assays. References: Leo O, et al. 1987 PNAS USA 84:1374 (for anti-CD3); Gross JA, et al. 1992 J Immunol 149: 380 (for anti-CD28)

Animals and other organisms

Policy information about studies involving animals; ARRIVE guidelines recommended for reporting animal research

Laboratory animals	Adult male and female HLA-DQ6.2 transgenic Ab0 NOD mice (3 months of age) were used. The strain #006023 had originally been purchased from Jackson Laboratories, Bar Harbor, USA. Details in housing are described in the manuscript.

Wild animals	This study did not include wild animals
Field-collected samples	No samples were collected in the field
Ethics oversight	Eläinkoelautakunta ELLA, Etälä-Suomen aluehallintovirasto (Board for Animal research ELLA, Southern Finnish State Administrative Agency, Finland)

Note that full information on the approval of the study protocol must also be provided in the manuscript.

Human research participants

Policy information about studies involving human research participants

Population characteristics	The T cell study included 28 pediatric Pandemrix-associated NT1 patients (5-20 years at vaccination, male and female, all DQB1*0602 positive) and 33 healthy Pandemrix-vaccinated control children and adolescents (4-16 years at vaccination, male and female, DQB1*0602 positive or negative). The plasma study included 37 pediatric Pandemrix-associated NT1 patients (4-16 years at vaccination, male and female, DQB1*0602 positive), 57 healthy Pandemrix-vaccinated control children and adolescents (0-14 years of age at vaccination, male and female, DQB1*0602 positive or negative), and 130 unvaccinated healthy control children and adolescents (male and female, DQB1*0602 positive or negative). Patients were defined based on the 3rd edition of the International Classification of Sleep Disorders. 3 sleep clinic patients without a diagnosis of NT1 were also included in this study (14-15 years at vaccination, male and female, DQB1*0602 positive).
Recruitment	Patients were recruited via the NARPANORD narcolepsy study. Patients were diagnosed in outpatient clinics at Finnish university hospitals, by pediatric neurologists, pediatricians or neurologists with expertise in sleep medicine. Patients were defined based on the 3rd edition of the International Classification of Sleep Disorders. Healthy Pandemrix-vaccinated controls (DQB1*0602 positive or negative; siblings of diabetes patients) were recruited via the Finnish Diabetes Registry. A selection bias can be ruled out.
Ethics oversight	The study protocol was approved by the Ethics Committee of the Hospital District of Helsinki and Uusimaa, Finland

Note that full information on the approval of the study protocol must also be provided in the manuscript.
